# Pelagic microplastics in the North Pacific Subtropical Gyre: A prevalent anthropogenic component of the particulate organic carbon pool

**DOI:** 10.1093/pnasnexus/pgad070

**Published:** 2023-03-09

**Authors:** Shiye Zhao, Tracy J Mincer, Laurent Lebreton, Matthias Egger

**Affiliations:** Research Institute for Global Change, Japan Agency for Marine-Earth Science and Technology, Yokosuka 237-0061, Japan; Harbor Branch Oceanographic Institute, Florida Atlantic University, Fort Pierce, FL 34946, USA; Department of Biology, Wilkes Honors College, Florida Atlantic University, Jupiter, FL 33458, USA; The Ocean Cleanup, Rotterdam 3014 JH, The Netherlands; The Modelling House, Raglan 3297, New Zealand; The Ocean Cleanup, Rotterdam 3014 JH, The Netherlands; Egger Research and Consulting, St. Gallen 9014, Switzerland

**Keywords:** microplastic, North Pacific Garbage Patch, biological carbon pump, vertical redistribution, marine particulate pool

## Abstract

Due to its ever-increasing ocean inputs, fossil-based microplastics (MP) comprise a considerable constituent in the particulate organic carbon (POC) pool, which is instrumental in ocean biogeochemical cycling. Their distribution within the oceanic water column and the underpinning processes, however, remain unclear. Here we show that MP prevail throughout the water column of the eastern North Pacific Subtropical Gyre, comprising 334 #/m^3^ (84.5% of plastic particles <100 µm), with exponential relationships between concentrations and water depth in the upper 500-m layer and marked accumulation below this layer. Our results suggest that the biological carbon pump (BCP) strongly contributes to the water column MP redistribution in terms of polymer type, material density and particle size, which in turn could influence the efficiency of organic matter export to the deep sea. We further show that ^14^C-depleted plastic particles predictably are an emerging nonneglectable perturbation to radiocarbon signatures in the deep ocean through depletion of the ^14^C/C ratio in the POC pool. Our data provide insight into vertical MP flux and highlight the potential role of MP in alternating the marine particulate pool and interactions with the BCP.

**Significance Statement**

The overall ubiquity and abundance of microplastics in the world's ocean make them an emerging constituent of the marine carbon pool. However, little is known about the dynamics of microplastics from the surface to the deep ocean. Here we present in situ evidence of microplastic particles throughout the water column of the North Pacific Garbage Patch located in the eastern North Pacific Subtropical Gyre, based on field-based measurements of their concentrations and chemical and physical properties. Our results imply that the biological carbon pump is a major contributor to the redistribution of microplastics in the ocean. ^14^C-depleted plastic carbon could bias radiocarbon measurements and geochemical estimates of particulate organic carbon.

## Introduction

Subtropical oceanic gyres occupying about 40% of the Earth's surface have been recognized as offshore convergence zones of buoyant plastics by previous modeling studies (1–5) and pan-oceanic quantitative studies (6–8). The current quantification of plastic pollution, however, predominantly focuses on the ocean surface layer (9), leaving plastic distribution within the ocean water column relatively unknown, thus impeding our understanding of ecosystem exposure to plastic pollution and of the ocean plastic mass balance. Furthermore, it limits mechanistic export models or evaluation of global effects through Earth system models (10, 11). This is largely due to the logistical and technological constraints of quantifying the vertical profiles of plastic in typically remote and often harsh ocean regions.

Recently, a few observations of microplastics (MP, <5 mm) from the surface to deep layers within offshore plastic accumulation zones have been conducted (12–15) (Table S1). Based on net-collected samples from the surface down to 2,000 m across the eastern North Pacific Subtropical Gyre (NPSG), a power law decline of plastic abundance (500 µm–5 cm) as a function of water depth has been identified (12). A similar power law decline for plastic >500 µm was also shown in the upper 300 m of the North Atlantic Subtropical Gyre (13). However, no such trend was found for the vertical distribution of MP (32–651 µm) in the upper 200 m of the water column along a north–south Atlantic transect based on analyses of in situ pump samples with micro-Fourier transform infrared (µFTIR) imaging (14). In the South Atlantic Subtropical Gyre, another study also revealed large quantities (up to 244 #/m^3^) of MP (20–321 µm) from in situ pump samples in surface to the near-bottom waters, with contrasting vertical distribution patterns of MP among stations. Concurrently, the abundances of large MP (>200 µm) captured by Manta net and MultiNet declined with water depth (15). A recent study (16) that collected plastics by deploying drifting sediment traps from 50 m down to 600 m in the North Atlantic Subtropical Gyre identified that the vertical flux of sinking MP (>10 µm) ranged from 0.12 to 1.7 mg m^−2^ d^−1^. Several models have been proposed to simulate MP dispersion in the ocean water column (17–20), but their results often do not match direct measurements of MP in the oceanic water column. For instance, these models predicted that buoyant MP in the world's ocean can only sink to certain depths (in the order of few hundred meters) (19); however, this does not adequately explain observations of buoyant MP in substantial abundances at the abyssal depths in the Arctic, Pacific, and Atlantic Oceans (12, 15, 21). Altogether, this highlights that more reliable and comprehensive quantifications of oceanic MP throughout the water column are urgently needed to more thoroughly characterize MP vertical distribution and to overcome significant uncertainties in predicting MP fate and impacts in the marine environment.

Dominating oceanic plastic particle counts (14, 15, 21, 22), MP at the lower microscale represent emerging anthropogenic particles of concern in the ocean, with particle abundances in some cases reaching up to 36,000 #/m^3^ as observed in the North Atlantic Subtropical Gyre (22). It is noteworthy that about 99% of plastics originate from fossil fuel carbon (23). The annual flux of this plastic carbon (plastic-C) into the global carbon cycle is roughly 280–360 million metric tons (24). Considering the continuous increase of plastic inputs and fragmentation in the marine environment (25, 26), substantial amounts of MP can be viewed as a consequential component of the marine particulate pool, which plays a vital role in ocean biogeochemical cycles (27). This could be particularly true for the oligotrophic subtropical oceanic gyres, due to the low rates of primary production in these waters and their central role in particulate matter export to the deep sea (28). For example, plastic-C of sinking MP (>10 µm) estimated from sediment trap samples comprised up to 3.8% of the particulate organic carbon (POC) measured concurrently at one station in the North Atlantic Subtropical Gyre (16). Knowledge of how anthropogenic plastic particles interact with naturally occurring particulate matter, however, is lacking. Such knowledge is imperative for understanding the immediate and long-term impacts of plastic pollution on the present and predicted future ocean biogeochemical dynamics.

**Fig. 1.**
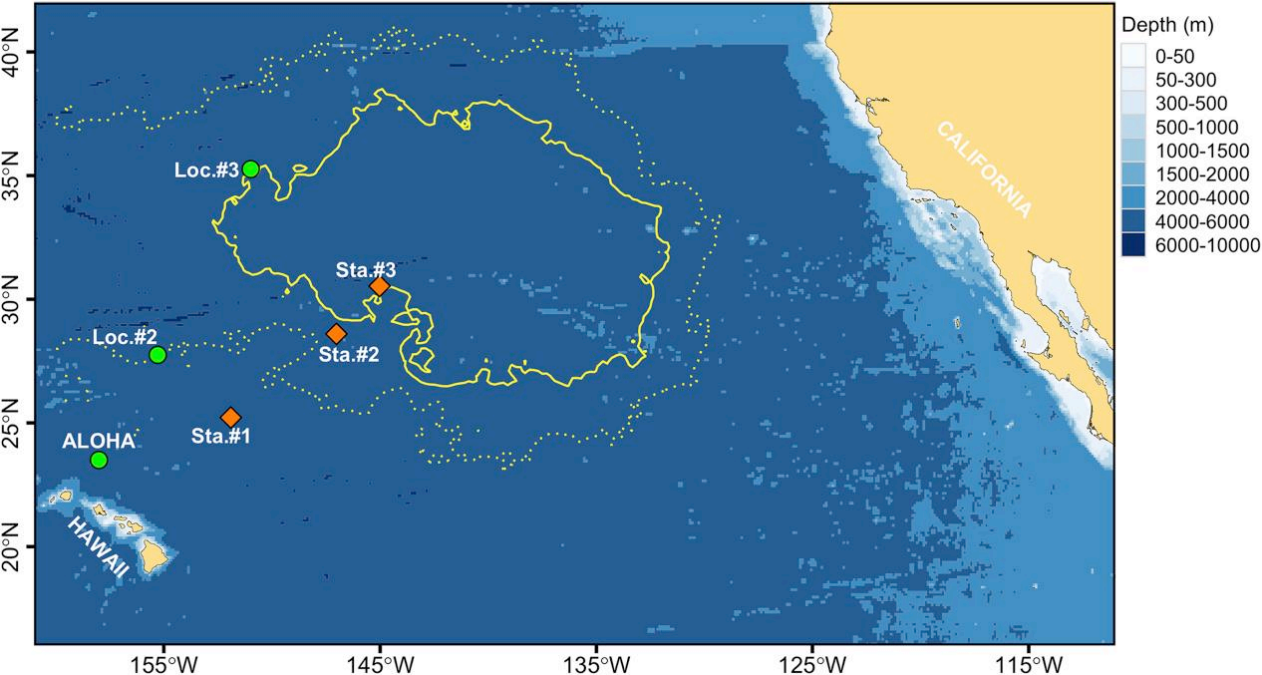
Map of the sampled area within the eastern NPSG. Diamonds represent stations (Sta. #1, Sta. #2, and Sta. #3) where in situ pumps were deployed to collect plastic samples in this study. Circles are locations taken from literature (ALOHA, Nov. 2018; Loc. #2 and Loc. #3 (29)), that measured the vertical profiles of particulate organic carbon. Solid and dotted lines represent the predicted inner (microplastic concentrations >1 kg/km^2^) and outer (microplastic concentrations >0.1 kg/km^2^) boundaries of the North Pacific Garbage Patch, respectively (30).

In this study, MP (>25 µm) collected via in situ pump filtration from 30 to 3,700 m depths at three stations in the eastern NPSG (Fig. 1) were quantified by automated interpretation of µFTIR imaging spectral data sets. Data from a companion study (12), concurrently measuring water column concentrations of large plastic particles (500 µm–5 cm) in net-collected samples at the exact same stations, were integrated to provide a holistic picture of the pelagic distribution of MP in this region. The in situ pump plastic-C was compared to in situ pump POC concentrations in the eastern NPSG (29) and POC measurements at Station ALOHA. Our results enable an in-depth analysis of MP export and fate in the NPSG, as well as on the interactions of MP with the biological carbon pump (BCP).

## Results

### MP abundances

**Fig. 2.**
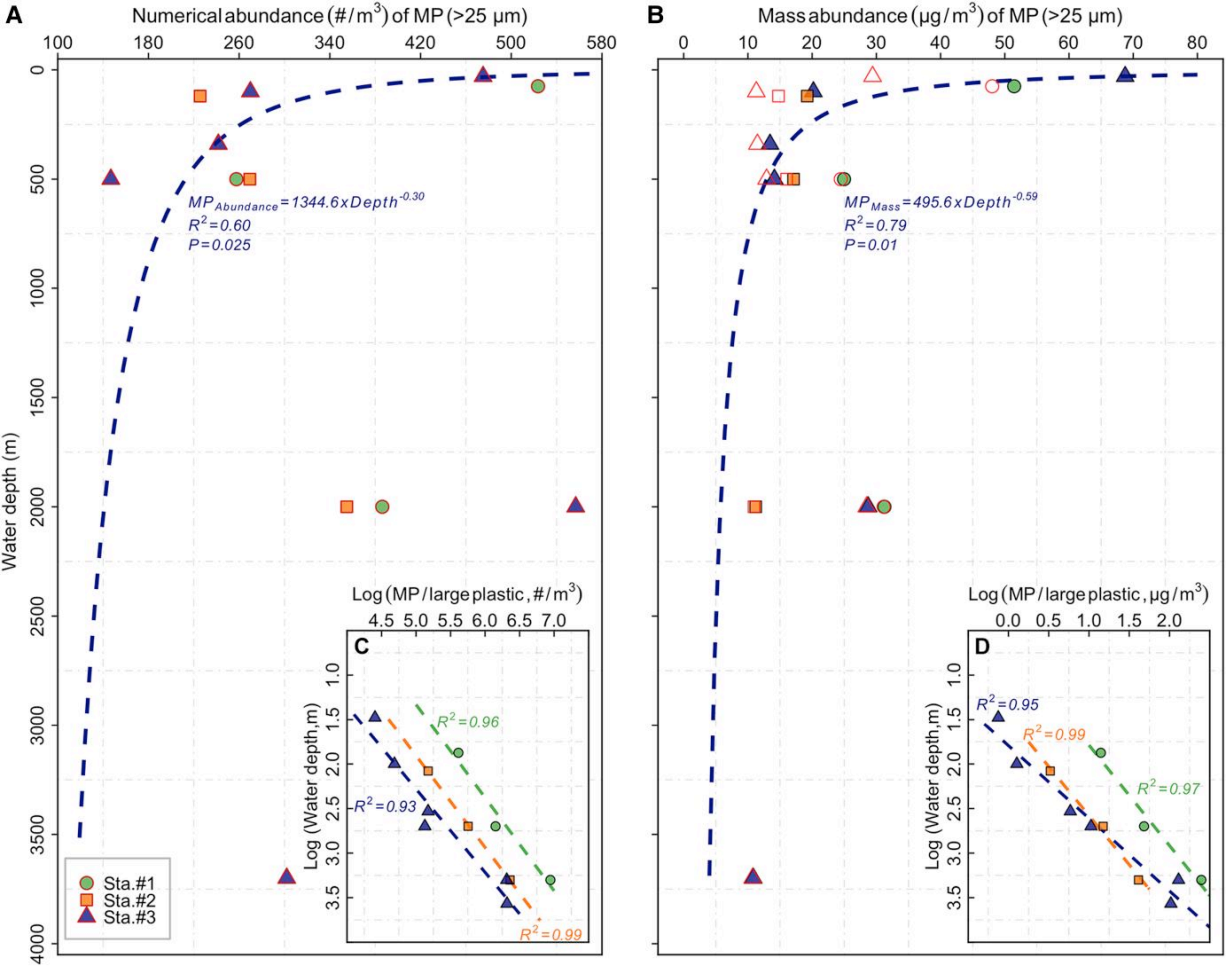
Vertical distribution of MP particles (>25 µm). The numerical A and mass B abundances of in situ pump-collected MP (>25 µm; over 99% of MP particles <500 µm) at three stations located outside (Sta. #1), in other boundaries (Sta. #2) and inside (Sta. #3) the North Pacific Garbage Patch (Fig. 1). Each filled shape is the sum of measured abundances of MP collected by the in situ pump samples in this study and predicted abundances of large plastic (500 µm to 5 cm) with the models provided in Egger et al. (12) (referred to as “total abundance” in the main text). Open shapes in Fig. 2A and B represent abundances in in situ pump samples only. The log–log plots of water depth vs. the ratios of numerical C and mass D abundances between MP (>25 µm; measured in pump samples) and large plastic [500 µm to 5 cm; predicted from Egger et al. (12)]. The dashed lines in Fig. 2A and B indicate the power law functions based on all the sum of abundances of plastic count and mass in the upper 500 m across all three stations. The colored lines in Fig. 2C and D represent the log–log regression fits between the ratio of MP/large plastic abundances and water depth. The corresponding linear regression equations are shown in Table S4.

Concentrations of polymer-specific MP are reported as particle number/mass per unit volume (#/m^3^ and µg/m^3^) in order to compare with previous studies. The filtered volume for each sample ranged between 397 and 510 L (Table S2), of which 100% were analyzed by µFTIR imaging. A total of 1,809 pieces of particles were assigned as plastic polymers by µFTIR spectroscopy, with 1,769 pieces in the samples and 40 pieces in the field blanks (Table S2). The averaged abundance of MP from in situ pump samples was 334 ± 128 #/m^3^ (20.8 ± 11.7 µg/m^3^), ranging from 147 to 557 #/m^3^ (10.7–48.0 µg/m^3^) (open red shapes in Fig. 2A and B and Table S3). The highest numerical abundance of MP was detected at 2,000 m at Sta. #3 (557 #/m^3^) and the lowest abundance at 500 m of Sta. #3 (147 #/m^3^). The mass abundance peaked at 75 m at Sta. #1 (48.0 µg/m^3^), while the lowest mass concentration was found at 3,700 m at Sta. #3 (10.7 µg/m^3^).

Within each station, the numerical MP (>25 µm) abundances captured by in situ pumps were four to seven orders of magnitude higher than concentrations of large plastic particles (500 µm–5 cm) at the same depths predicted according to the fitting models in Egger et al. (12) (Table S3). At each station, the ratios of numerical and mass abundances between in situ pump-collected MP (>25 µm) and predicted large plastic particles (500 µm–5 cm) showed log­–log linear relationships with water depth (Fig. 2C and D, Table S4). Summing numerical and mass abundances of plastic (in situ pump-collected MP plus predicted large plastic particles, i.e. 25 µm–5 cm) across the three stations revealed exponential declines in the concentrations of plastic count and mass in the upper 500 m of the eastern NPSG. Below 500-m water depth, plastic abundances no longer followed the prediction models and contained appreciably higher abundances than predicted (Fig. 2A and B).

### Polymer types and vertical connectivity

**Fig. 3.**
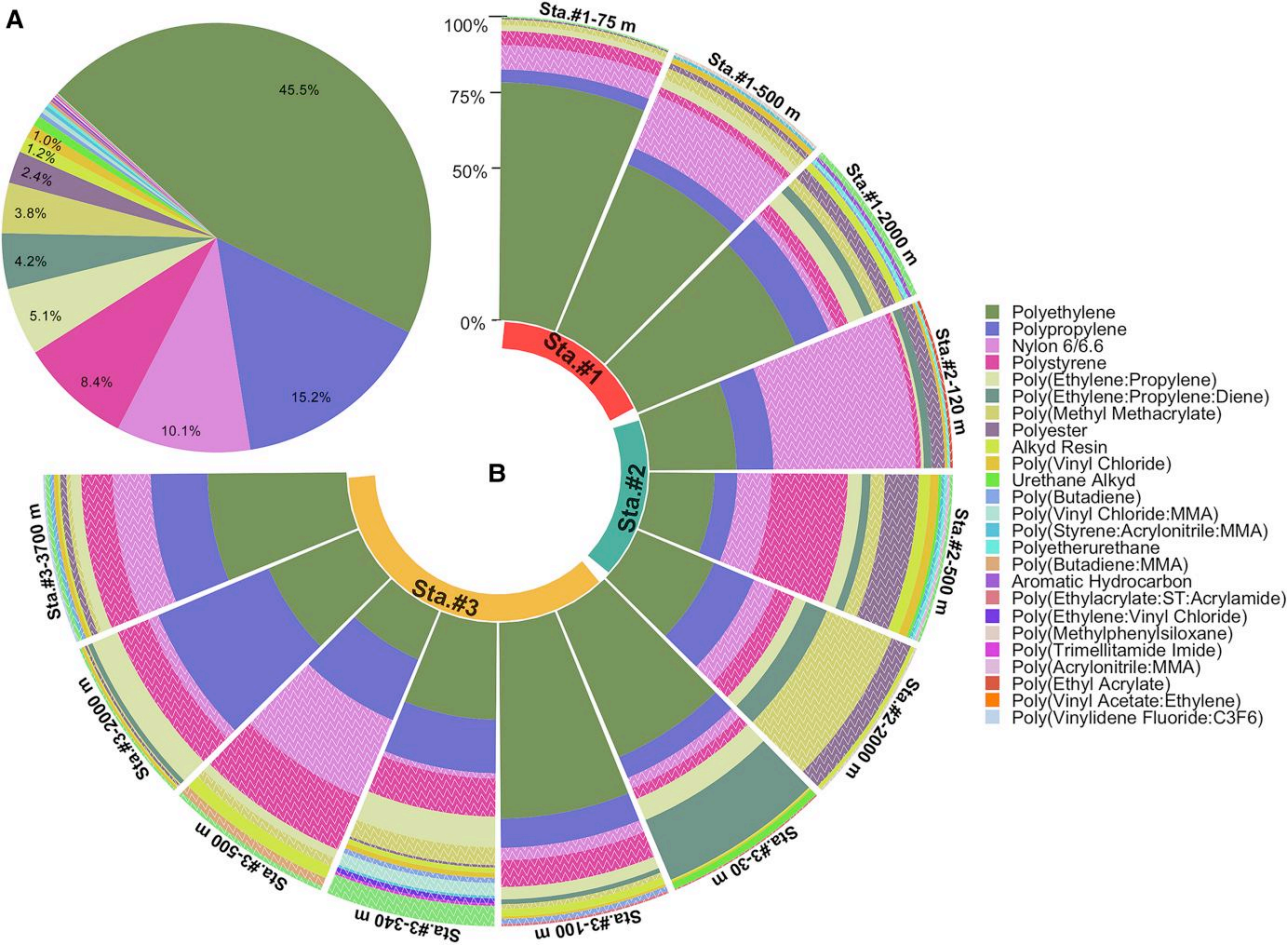
Proportions (%) of polymer types of in situ pump-collected MP particles (>25 µm). A The pie chart refers to the polymer composition for all samples. B The polar bar chart shows the relative composition of polymers for each station, different locations. The textured wave pattern in the polar bars represents the dense (density > 1.025 g/cm^3^) polymers.

**Fig. 4.**
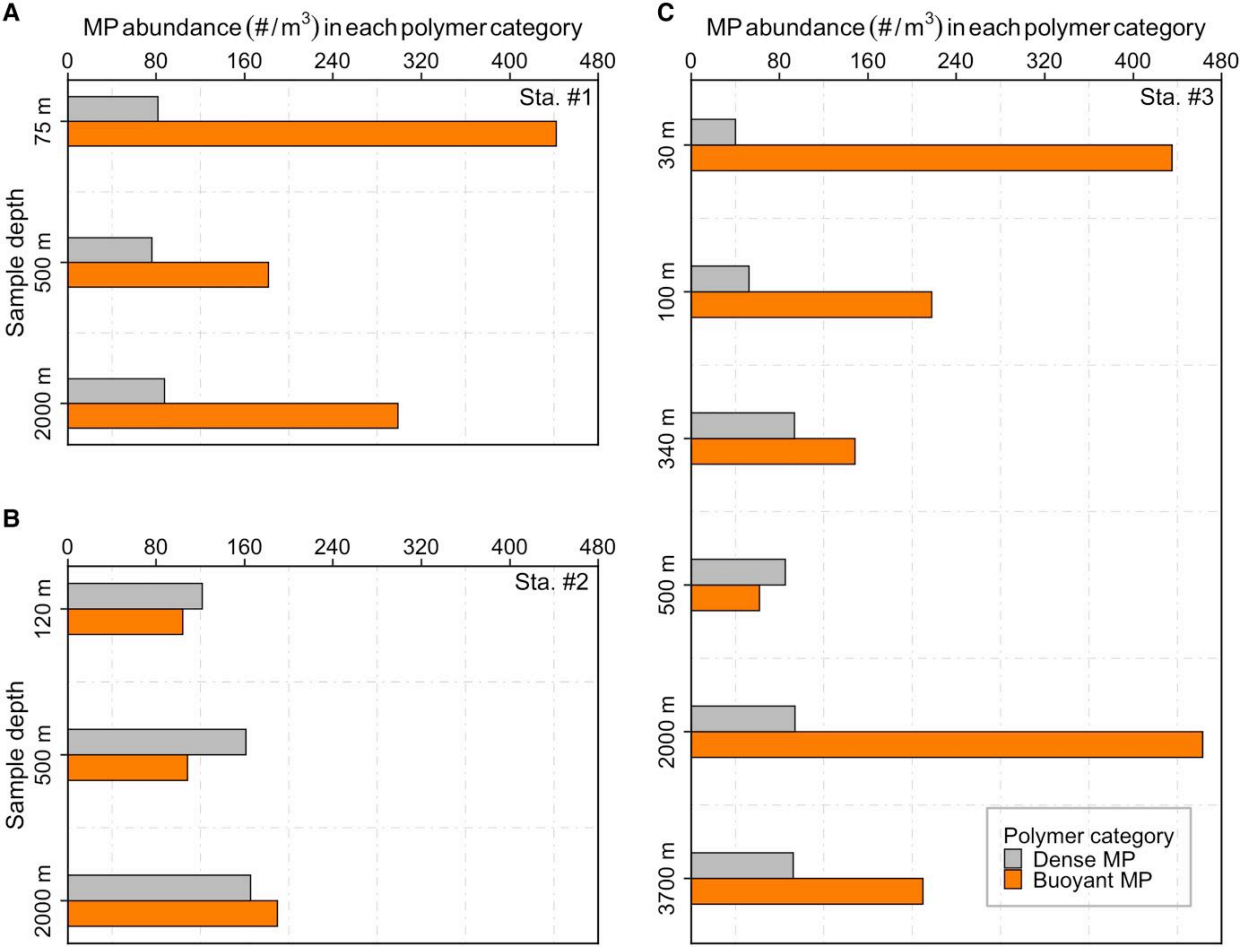
Numerical abundances (#/m^3^) of buoyant and dense MP at Sta. #1 A, Sta. #2 B, and Sta. #3 C. Dense MP have theoretical densities which are larger than 1.025 g/cm^3^. The densities of buoyant MP are <1.025 g/cm^3^.

A total of 25 different polymer types were confirmed in our pump samples (Fig. 3A, Table S5). Five polymer types [polyethylene (PE, 45%), polypropylene (PP, 15.2%), nylon 6/6.6 (10.1%), polystyrene (PS, 8.4%), and poly(ethylene-propylene) (PE-PP, 5.1%)] were detected from all depths of the pumped water samples, accounting for 60.3–97.2% of MP in each sample. A total of 71.2% (1,260 pieces) of the total particles (1,769 pieces) were buoyant plastics (*n* = 7 polymer types), e.g. having densities lower than seawater (*ρ* < 1.025 g/cm^3^), while the remaining 28.8% of particles (509 pieces) had a density higher than seawater (*ρ* > 1.025 g/cm^3^) (Table S5). The analysis of vertical connectivity of MP polymer composition showed that even though new polymers appeared continuously when moving from upper water masses to the next deeper one (Figure S1), polymers from all water masses and size fractions were principally dominated by polymers present in the North Pacific central water mass (CWM: 0–300 m). Additionally, the contribution of polymers in deeper layers, which were not present in upper waters, gradually increases from the small to the largest size fraction (Figure S1). At each station, numerical abundances of dense MP with respect to depth showed a lower variability compared to buoyant MP distributions (Fig. 4). Consequently, the coefficient variations of numerical abundances of dense (3.6–33.5%) plastics were considerably lower than for buoyant plastic polymers (35.0–61.0%) (Mann–Whitney–Wilcoxon test, *P* = 0.025, Figure S2).

### MP size

**Fig. 5.**
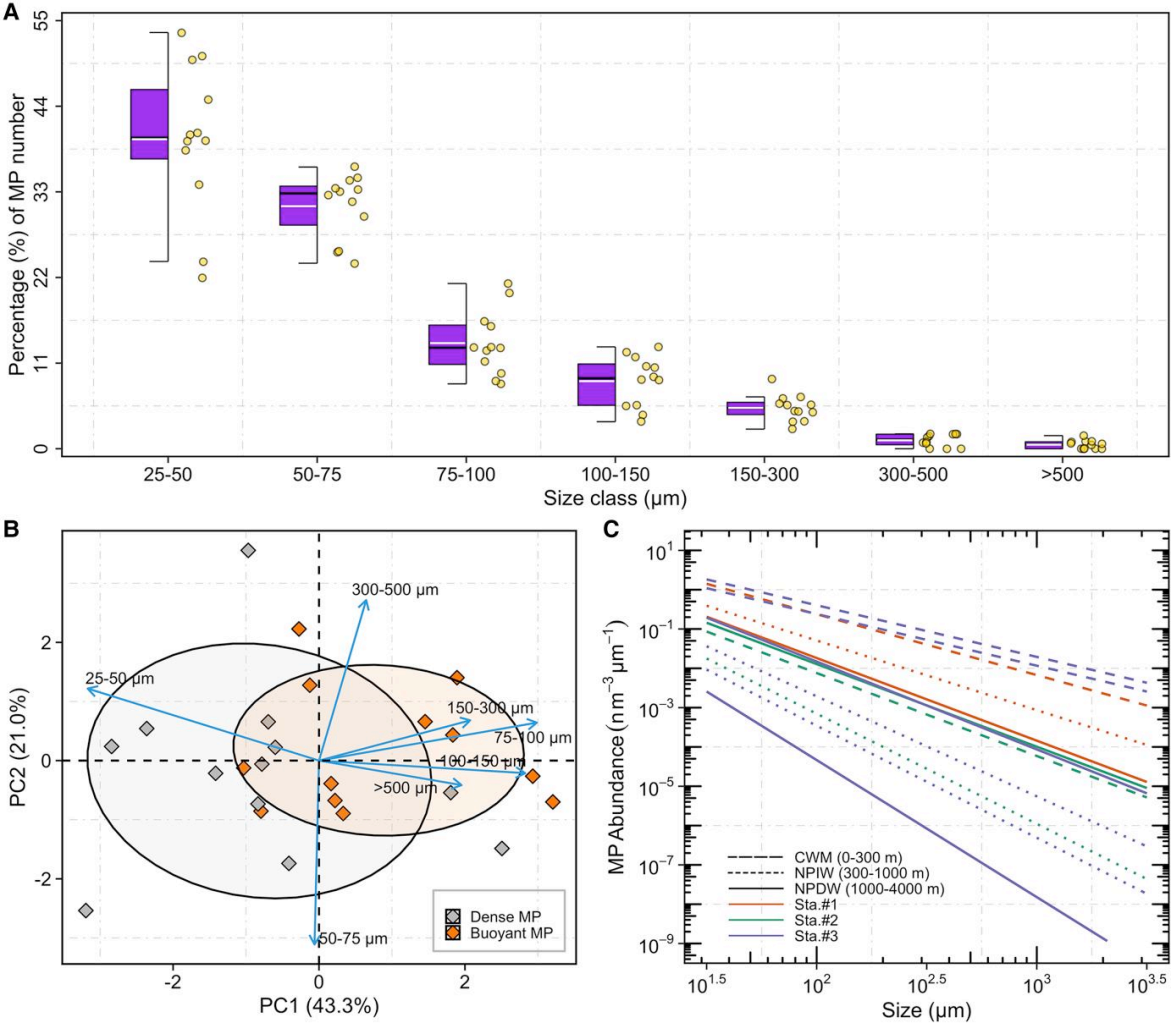
MP particles detected in in situ pump samples. A Box-and-whiskers plot of proportions of MP counts in different size categories from all pump samples. The black and white horizontal lines within the box represent median and mean values, and dots indicate individual in situ pump samples (*n* = 12). The top and bottom box boundaries represent the 25th and 75th percentiles; while whiskers indicate the largest and the smallest measured values within 1.5 interquartile ranges from the box. B Principal component analysis (PCA) biplot of the relative MP abundances (%) of seven size classes (25–50, 50–75, 75–100, 100–150, 150–300, 300–500, and >500 µm) with respect to dense and buoyant polymers. C Particle size distribution of pump-captured MP (particle length vs. particle abundance normalized by the width of seven size bins; see methods for details) for each depth at three stations. Lines indicate the fitted PSD slopes and intercepts. Dashed, dotted, and solid line types represent the PSD of pelagic MP within the three water masses of the eastern NPSG (CWM, 0–300 m; NPIW, 300–1,000 m; NPDW, 1,000–4,000 m), while different color lines indicate the PSD of MP at the three stations.

The high sensitivity of the µFTIR imaging allowed for the accurate identification of plastic particles ranging from 25 to 1,237 µm in our samples. MP particles in the range of 25–100 µm (*n* = 1,485) accounted for, on average, 84.5% of the total plastic counts (Fig. 5A and Figure S3). The relative abundances (%) of seven size classes of dense/buoyant MP were used to perform a principal component analysis (PCA). Dense plastics tended to cluster more toward small size fractions (25–50 and 50–75 µm) (Fig. 5B), while buoyant plastics were skewed toward larger size classes (>75 µm). Particle size distributions (PSD) were derived by fitting power law functions between the normalized abundances (particles m^−3^ µm^−1^) and MP length. The exponents (ξ) of PSD ranged from 1.31 to 2.85 and differed significantly across the three water masses (water mass pseudo-*F* = 33.7, *P*(perm) = 0.0087, Fig. 5C, Table S6). Lower PSD exponents were found in the top water layer (CWM, 0–300 m; ξ = 1.57 ± 0.38), compared to the North Pacific Intermediate Water (NPIW, 300–1,000 m; ξ = 2.49 ± 0.50), and North Pacific Deep Water (NPDW, 1,000–4,000 m; ξ = 2.27 ± 0.26). There was no significant difference in PSD slopes between three stations, although a slightly lower ξ was calculated for the measurements at Sta. #1 (1.81 ± 0.28) compared to measurements at Stat. #2 and #3 (ξ = 2.34 ± 0.4; ξ = 2.15 ± 0.68).

### ^14^C-Depleted plastic-C estimations

**Fig. 6.**
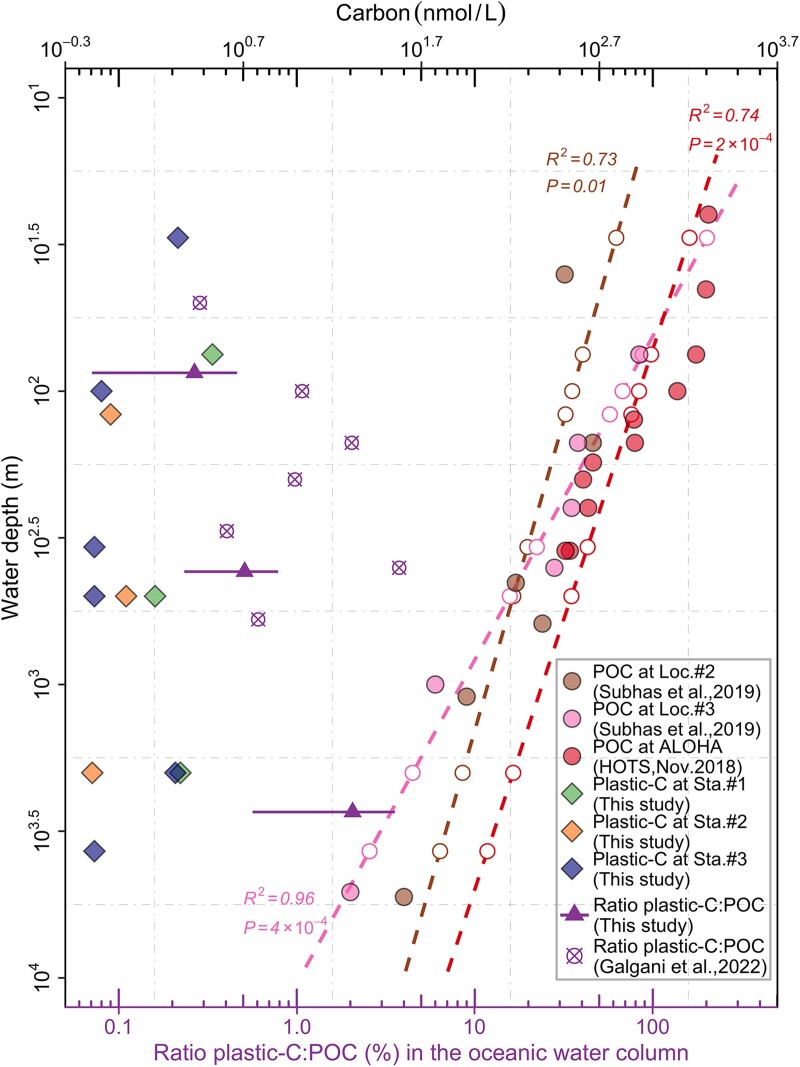
Comparison of estimated plastic-C (filled diamonds) collected on our in situ pump filters and particulate organic carbon (POC, filled circles) previously reported at three stations (ALOHA, Loc. #2, and Loc. #3) from the eastern NPSG (see Fig. 1. for geographic reference). The dashed lines represent the log–log linear regression fits between water depth and POC concentrations at these three stations. Regression parameters are also shown. Open circles indicate the predicted POC concentrations for water depths of our in situ pumps based on three linear regression models (Table S7). The purple triangles represent the averaged plastic-C/POC ratios (%, mean ± standard deviation) in the North Pacific CWM (average, 0.27%; median, 0.21%; range, 0.1–0.83%), NPIW (average, 0.51%; median, 0.45%; range, 0.16–1.0%), and NPDW (average, 2.1%; median, 1.47%; range, 0.43–5.0%). Crossed open circles represent ratios of plastic-C/POC directly measured in the sediment trap samples from the North Atlantic Subtropical Gyre by Galgani et al. (16).

The mean concentration of plastic-C collected on our in situ pump filters was 1.43 ± 0.86 nmol/L, spanning from 0.71 to 3.36 nmol/L (Fig. 6). Overall, differences between plastic-C and natural POC previously reported at stations close to our sampling sites decreased with respect to the water depth (Fig. 6). In the upper water column (CWM: 0–300 m), POC was approximately three orders of magnitude higher than plastic-C from similar depths. However, this discrepancy decreased by up to two orders of magnitude in the deep water mass below 2,000 m (NPDW: 1,000–4,000 m), where plastic-C could be equivalent to an average 2.1% (up to 5.0%) of POC in the eastern NPSG predicted based on the linear regression models of the logged POC measurements and water depths (open circles in Fig. 6 and Table S7).

## Discussion

### Vertical distribution of MP

**Fig. 7.**
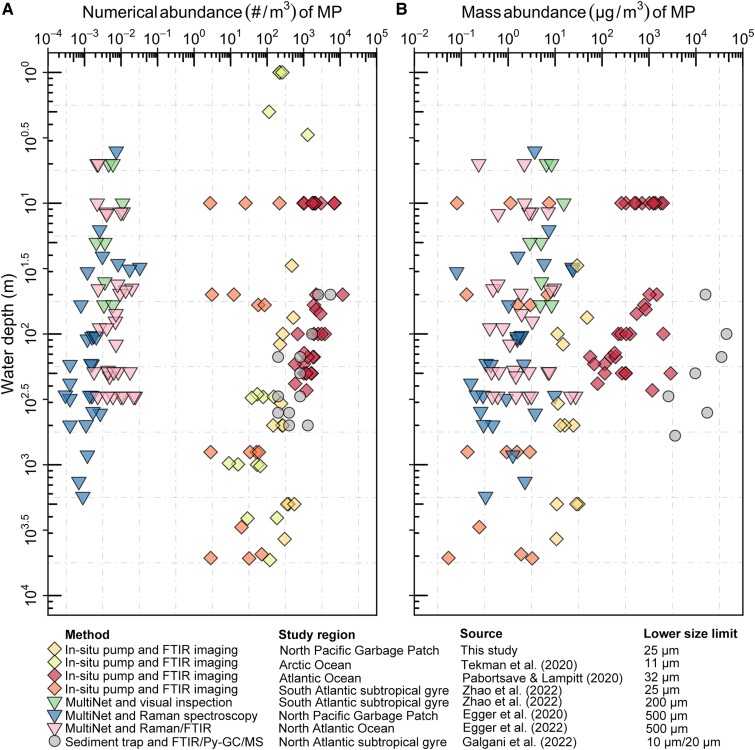
Comparison of the numerical A and mass B abundances of water column MP collected with the in situ pump, MultiNet, and sediment trap techniques at various study sites throughout the global oceans.

In this study, we present an updated comprehensive assessment of MP (>25 µm) pollution throughout the water column of the eastern NPSG. Our results reveal that average pelagic MP (>25 µm) abundance in the NPSG (334 ± 128 #/m^3^) is comparable to levels of MP (>11 µm) observed in the water column of the Arctic Ocean (161 ± 293 #/m^3^) (20) and generally around an order of magnitude higher compared with pelagic MP (20–321 µm) concentrations in the South Atlantic Subtropical Gyre (42 ± 54 #/m^3^) (15) (Fig. 7A, Table S1). Concentrations of MP (32–651 µm) reported in the upper 200 m along a latitudinal transect across the Atlantic Ocean by Pabortsava and Lampitt (14), however, are two orders of magnitude higher (2,272 ± 2,201 #/m^3^) than values measured in the South Atlantic by Zhao et al. (15) and about one order of magnitude higher than found in our samples from the eastern NPSG. With respect to the mass abundances of in situ pump-captured MP, our mass concentrations (11.5 ± 12.7 µg/m^3^) are somewhat higher than those observed in the South Atlantic Subtropical Gyre (2.0 ± 2.3 µg/m^3^) (15) but over 60 times lower than concentrations reported by Pabortsava and Lampitt (14) in the Atlantic Ocean (708.3 ± 690.3 µg/m^3^) (Fig. 7B). The large discrepancy between MP concentrations reported by Pabortsava and Lampitt (14) compared to values derived by Zhao et al. (15) and in this study could at least partly be due to different methodological procedures of the filter sample analyses. While we analyzed the entire in situ pump filter with µFTIR imaging in the present study, Zhao et al. (15) spilt ∼10% of the total particles of each in situ pump filter and identified these particles with µFTIR imaging; Pabortsava and Lampitt (14) estimated MP concentrations using a double extrapolation procedure based on filtering a split of 10% of the total particles on a 25-mm filter disc for polymer numeration and identification, followed by µFTIR imaging of 18% of the 25-mm filter area. It has been well documented that the theoretical extrapolations of the individual subsamples could introduce sever overestimation and underestimation of MP abundances of up to +600% (31, 32). Comparing plastic abundances in the current study to those presented in Galgani et al. (16) is challenging due to the distinct methodologies (Table S1). Our measurements are apparently lower than those detected in the sediment trap samples from the North Atlantic Subtropical Gyre (median numerical abundances, 200–800 #/m^3^; mass abundances, 0–44.9 g/L).

Our findings reveal that numerical concentrations of pelagic MP (>25 µm) collected by in situ pump are five to six orders of magnitude higher than previously reported numerical concentrations of large plastic particles (500 µm–5 cm) derived by underwater trawling at the exact same stations in the eastern NPSG (12) (Fig. 7A). Orders of magnitudes with higher abundance of in situ pump-collected MP (over 99% of MP particles <500 µm) compared to large plastic particles (500 µm–5 cm) are in good agreement with observations from the South Atlantic Subtropical Gyre (15). In contrast, mass concentrations for both in situ pump-collected MP (over 99% of MP particles <500 µm) and large plastic particles (500 µm–5 cm) in the NPSG water column are of similar order of magnitude, especially in the surface layer (Fig. 7B). Importantly, the ratios between the abundances (count and mass) of in situ pump-collected MP vs. large plastic (500 µm–5 cm) increase with water depth (Fig. 2C and D). This demonstrates that MP captured by in situ pump are becoming increasingly important at depth, in terms of both numerical abundance and plastic mass. Our data set indicates that utilizing either of the two sampling approaches for plastic budget calculations will result in substantial bias. Hence, a convergent approach is required to achieve reliable mass quantification of MP stocks and fluxes throughout the ocean water column. We herein conclude that the in situ pump approach is important for ecological risk assessments of pelagic MP pollution and that estimates of plastic mass loading in the ocean water column require both in situ pump and net-based sampling. The designed three baffle systems of the in situ pump employed in this study have been documented to efficiently reduce turbulence and minimize particle loss (33). However, it should be noted that partial loss of particles might remain during the operation of in situ pumps.

We further note that both numerical and total mass abundances of MP in the upper 500 m at all three stations (Fig. 2A and B) follow a classic pattern similar to that of sinking particulate matter in the global ocean (34–37). This concurrence strongly suggests that MP particles in the upper twilight zone (<500-m water depth) are linked to the ocean's BCP, which shapes the depth-dependent patterns of POC and transfers POC from the surface to the ocean's interior (35, 36, 38). MP incorporated into marine aggregates, an important component of the BCP (39), have been widely observed in field and laboratory settings (40–44). The attenuation with depth of natural particulate matter concentrations is mainly sustained by both biotic (metabolism by resident biota) and abiotic (mineral dissolution) processes in the upper 500 m (36). With respect to the anthropogenic MP particles, the abundance vs. depth relationship may be due to plastic oscillatory movement in the upper ocean. The resurfacing and sinking of plastics of varying densities (positive–neutral–negative buoyancy) are facilitated through interactions with a wide range of biogeochemical and physical processes. Of particular interest are the upper 500 m of the oceanic water column, where the highest POC removal occurs as a result of rapid biological consumption and remineralization of carbon (34, 37). Thus, MP in these water layers are likely to detach from marine aggregates through a combined processes of disaggregation, dissolution, solubilization, microbial decomposition, and biofilm removal (35, 45–47), resulting in buoyant plastics potentially regaining their buoyancy. High variability of buoyant MP abundances observed in our study may be due to, at least in part, positively buoyant plastics distributed along the depth continuum that are determined by superimposed factors (i.e. aggregations and polymer buoyancy) (Fig. 4 and Figure S2).

An intriguing feature in our data is that the numerical abundances of MP display subsurface accumulation at 2,000-m water depth (Fig. 2A), similar to previous observations in the Arctic Ocean and South Atlantic Subtropical Gyre (15, 21). It is possible that such a subsurface MP accumulation is attributed to processes related to the BCP (e.g. gravitational settling through aggregations and export by vertical migrations), through which MP overcome the “the oscillatory pattern” in the upper 500 m and, as a result, are transported downward to the deeper water layers. This hypothesis is further supported by the consistently higher abundances of buoyant MP at 2,000 m at all three stations (Fig. 4), which could only reach the deep ocean via incorporation into marine aggregates or vertical migrations of biota. In addition, the relatively slow current velocity may contribute to the increased abundances of plastic at depth as a similar MP hotspot has been observed at 4,800 m in the South Atlantic Subtropical Gyre where low-intensity currents were identified (15). This observation of MP accumulation in the deep ocean, however, is incongruent with the modeled vertical trajectory of MP (0.01–1 mm) in the NPSG (19) based on simulating biofouling and physical transport (i.e. advection, wind-driven mixing, tidally induced mixing), which predict that no plastic particles should reach below 300 m throughout the year. This discrepancy between model results and in situ observations indicates that the pelagic distribution in the NPSG water column is controlled by more complex biological and mechanical processes than currently resolved in numerical models.

Taken together, our findings suggest that certain plastic particles (especially buoyant plastics), which undergo both incorporation into and detachment from the BCP, are retarded in the upper portion of the mesopelagic zone, while plastic particles not undergoing these attachment–detachment cycles from the BCP are rapidly transported to greater depths (Figure S4).

### MP characteristics

The numerical predominance of MP less than 100 µm in our samples (84.5% of the total plastic pieces, Fig. 5A and Figure S3) is in good agreement with several previous research reports, which collected and concentrated particles on small pore-sized filters, followed by utilizing an automated FTIR imaging technique to detect plastics in different environmental samples (e.g. snow, fresh water, sediment, and seawater) (15, 21, 32, 48).

The polymer composition of MP particles in the NPSG water column was dominated by five polymer types (collectively ∼83.7% in count, Fig. 3A), i.e. PE, PP, nylon 6/6.6, PS, and PE-PP copolymer. Together, these polymers accounted for over 70% of the global plastic production in 2015 (49). The dominance of polyolefins (i.e. PE, PP, and PE-PP) observed in our samples is in good agreement with previous surveys in the eastern NPSG, which reported mostly PE and PP particles from the surface to deeper water layers in the region (12, 30). This result, together with the largely predominant CWM polymers (i.e. polymers first appearing in the water depth zone 0–300 m) throughout the water column (Figure S1; see Method “Vertical connectivity of water column MP”), strongly supports that the majority of MP at depths of the eastern NPSG originates from initially buoyant plastic debris accumulating in its surface waters (12). Notably, the polymer differentiation in a given size fraction in three water masses (CWM, 0–300 m; NPIW, 300–1,000 m; and NPDW, 1,000–4,000 m) declines with the decreased MP size, implying a more even distribution of smaller-sized MP in the water column (15). However, there was a larger contribution of polymers not present in the surface layer (CWM) in deep waters (NPIW and NPDW), especially in the large-sized fraction (Figure S1). This finding suggests that large-sized MP are more likely to reach the deep sea, which is particularly true for negatively buoyant plastic particles. For example, around 93% of MP particles (*n* = 68) only detected in the deeper water masses (NPIW and NPDW) are dense polymers (*ρ* > 1.025 g/cm^3^) (Figure S5).

Interestingly, we also discovered consistently less variability in numerical abundances of dense MP throughout the water column at all stations (Fig. 4 and Figure S2), indicating that they were relatively even distribution along the depth continuum. We mainly attribute this to the more stable sinking behavior of dense plastics compared to buoyant particles. A particle sinks as soon as its overall density exceeds its surrounding seawater density. Pristine dense MP with densities exceeding seawater density can only be redistributed in a unidirectional downward manner, neglecting ocean currents, waves, and the vertical migration of plastic via bio-uptake. Besides polymeric densities, particle size also plays a strong role in determining the sinking rates of MP (50). Our modeled settling velocities of the three most common dense MP particles in four size classes (25, 50, 75, and 100 µm), which are assumed to be colonized by a 5-µm-thick biofilm of diatoms, range from 7.0 × 10^−3^ to 1.45 × 10^−1^ m/day (Figure S6A). Coincidentally, we discovered that dense MP were skewed toward smaller size fractions (25–50 and 50–75 µm), compared to buoyant plastics (Fig. 5B). The plausible explanations to the observed smaller-sized dense MP may be the differences in physical, thermal, and mechanical properties of the polymer (51). For instance, the glass transition temperatures of dense polymers [such as Nylon (51°C), PS (90°C), and PMMA (90–110°C)], a key factor in determining the surface erosion rate of polymers, are usually higher than those of buoyant plastics [i.e. PE (−110°C)], which results in a higher probability of surface erosion and thus fragmentation into smaller particles (51, 52). Additionally, based on estimated sinking velocities, the time required for dense plastic particles to sink to 3,700 m (the deepest depth investigated in this study) may be from ∼70 (100-µm nylon particles) up to 1,418 years (25-µm PS particles) (Figure S6B). Compared to dense polymers, the buoyant plastics won't reach the ocean's depths in the same scenario. This implies that most of the MP found in our study are unlikely to have reached the deep sea by buoyancy forces only, considering that large-scale plastic production started only about 70 years ago (49). Our finding is corroborated by a recent study (16), which collected sinking plastics by deploying drifting sediment traps from 50 to 600 m in the North Atlantic Subtropical Gyre and suggested that plastics (10–100 µm) are most likely repackaged into marine snow aggregates, resulting in export of small MP to the deep ocean. Although particle export is largely attributed to the gravitational settling (the functioning of the BCP), additional export pathways (i.e. the physical subduction and active transport by vertical migrants) also considerably contribute to the delivery of particles to deep waters, which act on all particles from suspended to sinking. The latter pathways occur concurrently with the BCP and thus overlap between these export mechanisms, consequently accelerating the rates of particle export (53). Taking all this into account, we conclude that the downward transport of MP (numerically dominated by particles <100 µm) mainly depends on the nonlinear interactions among different processes, such as the BCP and other physical processes.

PSD representing the relationship between the size of particles and their concentrations has been widely used to characterize marine particles (54–56). The significantly lower PSD slopes of MP in the CWM (ξ = 1.57 ± 0.38) compared to in the deeper water layers (NPIW, ξ = 2.49 ± 0.50; NPDW, ξ = 2.27 ± 0.26) suggests an underabundance of small-sized fractions of plastic particles in the upper water layer (Fig. 5C), thus supporting a previously postulated size-selective transport of MP particles in the water column (6, 57). PSD slopes of MP particles from the outside to the inside of the North Pacific Garbage Patch exhibit slightly different distributions (Fig. 5C and Table S6). The steeper PSD slopes inside the accumulation zone (Sta. #2 and Sta. #3) suggest a greater proportion of smaller particles in these oligotrophic waters. This observation is in line with the reported increase in the relative abundance of floating MP from the outside to the inside of the North Pacific Garbage Patch (58). This observed trend further agrees well with a previous survey on natural particles, of which the PSD slope progressed from lower near the coast to higher in the North Atlantic Subtropical Gyre (59).

### Plastic-C and its potential for skewing POC measurements

The increase in the ratio of plastic-C to POC with increasing water depth, from an average of 0.27% in the CWM (range, 0.1–0.83%; median, 0.21%) and 0.5% in NPIW (range, 0.16–1.0%; median, 0.45%) to 2.1% in NPDW (range, 0.43–5.0%; median, 1.47%) (Fig. 6) is indicative of the recalcitrance of polymeric C relative to organic C. More notably, this elevated plastic-C:POC ratio (up to 5%) in the deep sea suggests that Δ^14^C-depleted plastic-C could become a nonneglectable fraction of the POC pool, particularly in waters with high plastic loading and low POC concentrations. The increased plastic-C:POC ratios were comparable to those reported by Galgani et al. (16) (averagely 1.1%, ranging from 0 to 3.8%), who estimated the plastic-C and directly measured the total POC captured in the sediment traps deployed between 50 and 600 m in the North Atlantic Subtropical Gyre. Moreover, the authors identified a general increase of plastic-C:POC ratios with increasing water depth (Fig. 6) (16). Radiocarbon (^14^C) has been widely used to investigate the carbon biogeochemistry of bulk marine organic carbon reservoirs such as POC and dissolved organic carbon (60). Altogether, substantial ^14^C-depleted plastic particles added to the natural POC pool could be expected to hamper ^14^C-based dating. For example, on average 2.1% isotopically depleted plastic-C observed in the deep water (NPDW) will make the POC samples appear to be about 168 years older than they truly are. As plastics entering the ocean will continue to increase in the upcoming years, ^14^C-depleted plastic particles will work as a new type of fossil carbon-based perturbation (61) to radiocarbon dating through lessening the fraction of radioactive ^14^C in the marine particulate pool. It should be noted that the carbon values of natural organic particulates used to estimate the plastic-C:POC ratios in our study were derived from the literature rather than direct measurements of our in situ pump samples. However, the trend of POC in the eastern NPSG is relatively consistent (Figure S7). Furthermore, POC values at Sta. ALOHA are generally higher than those at Loc. #2 and Loc. #3 (Figure S8), which are situated inside the North Pacific Garbage Patch and are expected to have high plastic concentrations (30). This indicates a high plastic-C and low POC pattern at Loc. #2 and Loc. #3. Therefore, the averaged POC value at Sta. ALOHA, Loc. #2, and Loc. #3, which are used to predict the plastic-C/POC ratio, should provide fair estimates to evaluate the influence of plastic-C on the natural particulate organic pool. However, we still emphasize that the current estimate of the plastic-C/POC ratios serves as a benchmark for future studies investigating the impact of plastic-derived carbon on the marine POC pool, and further efforts to simultaneously quantify plastic-C and POC are merited. Additionally, the stable carbon isotopic composition (δ^13^C) of particulate organic matter is widely used for the source characterization of organic matter. Typically, δ^13^C of marine-sourced organic matter is higher than that of terrestrial organic matter (62). Fossil fuels, which are ancient plant and animal material, are depleted in ^13^C because plants prefer to take up ^12^CO_2_ over ^13^CO_2_ (63). Therefore, the presence of fossil carbon-based plastics in marine particulate pool could lower the δ^13^C values and thus disturb the source attribution of organic matter.

### Implications for the BCP

The omnipresent MP particles with highly diverse chemical natures from surface to bathypelagic waters (3,700 m) could have important implications for the functioning of pelagic food webs and the transport of particulate organic matter to the deep sea, thus ultimately affecting the efficiency of organic carbon sequestration in the deep ocean. Such high amounts of prey-sized plastic particles (ranging from 147 to 524 #/m^3^) in the eastern NPSG interior may favor a high plastic-to-organism ratio, subsequently causing increases in the probability of encounter and ingestion by marine biota. For example, the maximum abundances of major particle feeders (poecilostomatoid and harpacticoid copepods, salps, polychaetes, and ostracods) in the epipelagic (upper 200 m) and mesopelagic (200–1,000 m) waters at ALOHA, were ∼132 and 16 individuals/m^3^, respectively (64). MP particles at the similar depths in our study outnumber particle feeders by two to four times in the epipelagic zone and by 9–17 times in mesopelagic zone. Once ingested, MP and plastic-associated chemicals may adversely impact the health of zooplankton (65, 66) and potentially alter their community structure and thus the flux of organic matter to the deep ocean.

MP incorporated into marine aggregates, a key component of the BCP, could impact the export of organic carbon (67–71). Laboratory experiments indicate that incorporation of buoyant MP (i.e. PE and PS) apparently reduced the density of marine snow and copepod fecal pellets, thus resulting in slower sinking rates. The predominance of buoyant MP throughout the eastern NPSG water column therefore indicates a potential reduction in the settling velocities of particulate matter. Slowing sinking particles potentially increases the retention of marine aggregates within the ocean's twilight zone (38) and thus increases attenuation (microbial consumption, physical disaggregation, and zooplankton grazing) of aggregated particulate organic matter. Together, this could reduce the transfer of organic matter into deeper waters. Moreover, a laboratory study showed fecal pellets containing PS plastics are prone to lose their structural integrity compared to fecal pellets devoid of plastics (72), which potentially exacerbates their negative influences of MP on the transfer efficiency of the BCP.

In summary, high abundance of MP (147–557 #/m^3^; 10.7–48.0 µg/m^3^) identified in our in situ pump samples shows that the ocean interior, Earth's largest biome, is a critical sink for plastic debris, conforming to previous surveys (12, 14, 15, 21). The role of MP particles (over 99% of MP particles: <500 µm) collected by in situ pumps and the burden they inflict on the ocean, in terms of both particle count and mass, becomes more pronounced in the deep sea (Fig. 2). The BCP, together with particle properties (i.e. size and buoyancy), contributes to the observed distribution patterns of the water column MP (Fig. 2). The presence of MP particles with different size, buoyancy, and chemical composition in aggregates and fecal pellets has the potential to reduce the vertical transfer efficiency of particulate organic matter, meriting priority for future research. Additionally, such information is becoming increasingly invaluable for the study of the validation and testing of different models of plastic flux and of impacts of plastic pollution on ecological health and biogeochemical cycling (10, 11, 73). Furthermore, our results indicate that Δ^14^C-depleted plastic particles could be an emerging nonneglectable perturbation to the radiocarbon dating in the deep ocean through isotopic dilution of the particulate pool and result in an overestimation of the natural POC in the high plastic-laden environments, as well as biasing the δ^13^C values (Fig. 6). Further efforts are needed to reliably quantify MP counts and masses through the oceanic water column over seasons at regional and global scales, aiding in the mechanistic understanding of the flux of plastic particulate matter into the ocean's interior and the interactions between plastic pollution and the BCP. The inevitable increase in the amount of plastic particles in different sizes and polymeric compositions throughout the oceans (25, 26) will undoubtedly enhance the role of plastic particles in the marine particulate matter pool. Therefore, acknowledging plastic particles as an emerging anthropogenic component of the particulate marine reservoir and adopting biogeochemical perspectives and techniques will enhance our knowledge of plastic debris and its risk in the ocean system.

## Materials and methods

### Sample collection

Particulate samples were collected aboard the Maersk Transporter in November–December 2018 at three research stations (Sta. #1, Sta. #2, and Sta. #3). Sampling stations were selected to represent areas outside of, at the edge of, and inside the North Pacific Garbage Patch as predicted by Lebreton et al. (30). Sampling locations are shown in Fig. 1. At each station, a profile including salinity, temperature, dissolved oxygen, and fluorescence was conducted from the surface to the deepest sampling depth using a conductivity-temperature-depth probe (Sea-Bird Electronics, SBE-5 T). At each station, a McLane Research in situ pump with two flow paths large volume water transfer system (WTS-LV) was deployed to the predetermined depths (Table S2) for ∼1 h to capture both suspended and sinking particulates (33, 74). Each flow path was equipped with a 142-mm-diameter filter holder containing antiwashout baffle systems to prevent particle loss. Particulates in the water were concentrated onto precleaned 142-mm stainless steel (SS) meshes with an aperture size of 10 µm (Lorentz Mühlenbau GmbH, Germany). Filtration volumes were measured with the flowmeters on the McLane WTS-LV. Field blank filters (142-mm SS meshes; *n* = 5) were simultaneously deployed with the in situ pump at certain depths of each station (Table S2) by specially disconnecting one of the dual filter holders from pumped water flow. Immediately upon retrieval, the pump heads were transferred to the onboard laboratory, where the SS meshes were carefully removed from the pump heads and stored frozen (−20°C) in glass petri dishes until further analysis onshore. On basis of the hydrographic properties within the sampling depths (salinity and density, Figure S9), the final data set of in situ pump samples (*n* = 12) was obtained from three distinct water layers: North Pacific CWM (0–300 m), NPIW (300–1,000 m), and NPDW (1,000–4,000 m) (75).

### MP extraction

In the onshore laboratory, any visible plastic-like particles were picked out from the filters with clean forceps under a stereomicroscope and saved for polymer identification. Note that fibrous particles were also picked out from the filters and were excluded from this study due to possible contamination of the samples by airborne fibers during sample collection (no laminar hood was available at sea). The remaining particulates on each filter (from both samples and field blanks) were rinsed off with 300-mL filtered 70% ethanol and subsequently collected into a precombusted glass flask. This was repeated three times to collect all particles in the beaker. The rinse solution was heated briefly at 40°C to evaporate the ethanol. Then, the sample was incubated with 20 mL of 5% potassium hydroxide (KOH, Fisher Scientific) at 50°C for 72 h, followed by the neutralization with hydrochloric acid (HCl, Technical grade, Fisher Scientific, USA). The solution was transferred into two to three precombusted 20-mL scintillation vials (20 mL, DMK, Life Sciences Kimble). Based on the volume, the solution with particles were homogenized by gentle shaking, evenly split into three aliquots and filtered on three Anodisc filters (0.2-µm pore size, GE Whatman) through a glass filter holder (Advantec, 13 mm in diameter). Anodisc filters (*n* = 3) for each sample were saved in a precombusted glass petri dish and dried at 37°C overnight for µFTIR analyses.

### MP identification by µFTIR

The visible particles were identified with the FTIR spectrometer (Thermo Scientific Nicolet iN10, USA) in the attenuated total reflection mode in the spectral range from 3,600 to 1,250 cm^−1^. The FTIR spectrometer worked in a high-efficiency particulate absorbing (HEPA) filtered laminar flow hood. The chemical nature of particles on Anodisc filters (in total: 55 filters) was confirmed with the µFTIR imaging method by Zhao et al. (15). Briefly, the entire Anodisc filter (filter area = 133 mm^2^ in diameter) was scanned using the FTIR microscope in transmission mode using an MCT/A detector (aperture size, 25 µm × 25 µm, 1 scan at 16 cm^−1^ resolution; step size, 21 or 22 µm). The generated spectral (∼16 million individual spectra) were analyzed via two-step confirmation: (i) each spectrum in the data sets was compared automatically against a transformed reference library with the search algorithm (Pearson's correlation) in Python. When Pearson's correlation coefficient was larger than 0.8, the position (*x* and *y* coordinates on the chemical map) and polymer types of each identified spectrum were recorded; (ii) the selected spectra were tracked in chemical maps, and the polymer identity was confirmed again by comparing with the Hummel Polymer Sample Library in OMNIC Picta (Thermo Scientific). The software interpretation was systematically validated (>70% match) or rejected (<60% match). Spectra with a match between 60 and 70% were individually reinterpreted by confirming the clear presence of specific polymer peaks. The particle was only regarded as plastics if its spectrum was confirmed as the same polymer by both steps. For further details on the methodologies, the readers are kindly referred to Zhao et al. (15). Polymers with theoretical densities lower than 1.025 g/cm^3^ are regarded as buoyant plastics, while polymer species (*ρ* > 1.025 g/cm^3^) are referred to as dense plastics.

### MP size and volume

Based on the coordinates of the chemical map generated by scanning the respective Anodisc filter, each identified particle was located on the chemical map. The major (the maximum Ferret's diameter) and the minor axis (the longest axis perpendicular to the major axis) were measured on the chemical map with the ruler tool in the OMNIC Picta software, according to methods described in Zhao et al. (15). The average difference between Ferret's diameters of MP (129 pieces) as measured on infrared images compared to those on the concurrent optical images was 4.7 µm (median 4.5 µm, Figure S10). On basis of previous studies (76, 77), each MP was assumed to be an ellipsoid to estimate its volume using a modified method. The intermediate axis (*c*) of each MP was estimated by multiplying the median minor axis of all identified MP pieces and the ratio minor/major axis of all particles. Based on the ellipsoid volume model, the volume (*V*) of MP was calculated using the major (*a*), minor (*b*), and intermediate (*c*) axis:


(1) $$V_{\mathrm{MP}}=\pi abc/6$$

Previous surveys imply that most net-collected MP fragments (>300 µm) are flat (dimension: 2–3) and irregular in shape (30, 78). It has been demonstrated that reported methods studies (76, 77) produce substantial geometric biases, especially for larger particles (79). Contrastingly, our method facilitates conservative estimates of the third axis, thus minimizing the overestimation of volume and mass.

### MP mass and estimation of ^14^C-depleted plastic-C

The mass of MP was calculated by multiplying particle volume (*V*) with the density (ρ, Table S3) of the identified polymer. The carbon mass of MP particle was determined by using the portion (C%) of carbon in the chemical formula of each polymer (Table S5):


(2) $$\;C_{\mathrm{plastic}}=\rho V_{\mathrm{Mp}}C\text{\%}$$

Out of 25 polymer types confirmed in this study, the chemical formulae of 12 plastic types were found, accounting for 96% of the total MP counts. The median carbon portion (62.5%) of these 12 polymers was applied to the remained 13 plastic types, of which the chemical formulas were not identified (Table S5).

### Particle size distribution

The formulation of the power law model which is used to approximate the PSD is


(3) $$n(D)=n_{0}{\left(\frac{D}{D_{0}}\right)}^{\xi }$$

in which *n* (particles m^−3^ µm^−1^) is the particle abundance normalized by the logarithmically width of each size bin (25–50, 50–75, 75–100, 100–150, 150–300, 300–500, and 500–5,000 µm); *D* (µm) is the geometric average of the two boundaries of each class bin (80); and $D_{0}$ (µm), the reference diameter, is 35.4 µm, generated from the smallest size bin in our fit. $n_{0}$ (particles m^−3^ µm^−1^) is the normalized abundance at $D_{0}$, and ξ is the exponent of the power law (81). The exponent (ξ), also referred to as the PSD slope, provides information on the relative abundance of small to large particles: the proportion of smaller particles increases with the PSD slope (54) and can be utilized to compare the PSD slopes across environments (59).

### Predicted large plastic abundances

After the deployments of the in situ pump to collect water column particulate samples were finished, net-based techniques were utilized to sample for large plastic particles (500 µm–5 cm) from the surface to 2,000-m depth at the same stations (Sta.#1, Sta.#2, and Sta.#3). For this, a Multiple Opening and Closing Net with an Environmental Sensing System (MOCNESS) was deployed to collect samples from 2 m down to 2,000 m. Concurrently, a manta trawl (Ocean Instruments, Inc.) was deployed for sampling plastic particles afloat in the surface water (upper 15 cm). The data set of the net-collected large plastic has been published in Egger et al. (12) and revealed log–log linear correlations between the abundance of large plastic (both particle count and mass) and water depth at each station. These power law functions reported in Egger et al. (12) were used to estimate the numerical and mass abundances of large plastic at the same depths and locations where the in situ pump was deployed. Total abundances were subsequently calculated by summing the predicted large plastic abundances (net-based samples) and measured in situ pump-captured MP abundances at the same depths (Table S3). This total abundance is able to provide a more complete view of MP inventory in the eastern NPSG interior, ranging from 25 µm to 5 mm in size.

### Comparison of POC and ^14^C-depleted plastic-C

Nonliving POC measured at two stations (Loc. #2 and Loc. #3, Fig. 1) in the eastern NPSG (29) was used to compare the estimated plastic-C captured by in situ pumps. To measure the POC, Subhas et al. (29) filtered particulate samples on the Advantec GC-50 glass fiber filters (0.5 µm pore size) at six depths (from 40 to 5,300 m) with the dual-flow path in situ pump (WTS-LV, McLane Research Laboratories, Inc.) in August 2017. POC was calculated by the difference between the total carbon and particulate inorganic carbon (PIC) of the in situ pump filters. For more details on the method, the reader is referred to Subhas et al. (29). Additionally, POC at ALOHA station (158°W, 22.75°N) was calculated by multiplying the total particulate carbon (TC = POC + PIC) by the empirical POC:TC ratio (∼90%), which was measured at Station ALOHA (35, 82). The data of TC at Station ALOHA were from bottle samples at 10 different depths (from 5 to 350 m) collected on 2018 November 16 during the cruise KM 18–21 (http://hahana.soest.hawaii.edu/hot/hot_jgofs.html). These data revealed a power law relationship between water depth and POC at stations Loc. #2, Loc. #3, and ALOHA (Table S7), which we used to estimate POC concentrations at our sampling depths and to subsequently compare these values to estimated plastic-C concentrations at the same depth.

### Vertical connectivity of water column MP

To determine the vertical connectivity of MP types, we explored whether specific polymer types present at one depth could be found in the other depths. To do so, all polymer types were categorized into three water mass groups: CWM (0–300 m), NPIW (300–1,000 m), and NPDW (1,000–4,000 m), defined by the water mass where they were first identified, assuming a directionality from surface to bathypelagic waters and considering all stations together. For example, if a polymer type was detected in any of the upper water mass, it was categorized as CWM, but if a polymer type was first detected in middle water mass but not in the former water mass (CWM), it was categorized as NPIW and so on.

### Modeled sinking velocity of dense MP colonized by biofilm alone

To estimate the sinking velocity of negatively buoyant plastic particles (having equivalent spherical diameters 25, 50, 75, and 100 µm) colonized by diatom biofilm, the model described in Kooi et al. (46) was applied to both spherical and nonspherical particles (46, 50). For the modeling, we selected three plastic polymer types which accounted for 78% of the dense MP counts (509) in our study [e.g. nylon, PS, and poly(methyl methacrylate)]. The density of diatom biofilm used in our study is 1.15 g/cm^3^ referring to the observed values (83). Our model assumes that each particle is fully covered by 5-µm-thick diatom biofilm, which theoretically accommodates approximately two layers of these 3-µm-thick diatoms according to the measured diatom cell dimensions (L × W × H: 12.5 × 4.5 × 3 µm) (79).

### Contamination prevention

To minimize contamination with plastic fragments in the field, standard nonplastic laboratory and fieldwork equipment such as metal and glass was used whenever possible, and the samples and pump filter holders always remained covered with aluminum foil when not in use. All filters were carefully inspected for cleanliness under a microscope prior to heading offshore and stored individually in clean glass petri dishes. At sea, the filter holders were rinsed and filled with Milli-Q water filtered through 10-µm SS filters in the fieldwork laboratory. Upon retrieval, the filters were immediately transferred back the glass petri dish and stored frozen in the dark.

In the onshore laboratory, measures proposed in Zhao et al. (41) were adapted during processing of the samples. Briefly, all liquids used in this study (including Milli-Q water, ethanol, KOH, and HCl) were passed through precombusted 0.7-µm GF/F filters (Whatman). All glassware (e.g. beakers, filtration system, petri dishes, and Pasteur pipettes) were rinsed with Milli-Q water and wrapped with aluminum foil. Glassware and GF/F filters were combusted at 450°C for 8 h to ash any organic materials. All sample handling and sample identification with µFTIR imaging were conducted under a clean laminar flow cabinet (HEPA filter). Nitrile gloves and cotton lab coats were worn during the laboratory activities.

Three procedure blanks were also performed to account for the potential contamination in the laboratory (Table S2). For each station, a procedure blank was performed by exposing a precombusted 25-mm Petri dish to the air under the laminar flow cabinet. Then, the potential particles collected in the petri dish were processed following the identical procedure as for the pump samples and scanned with the µFTIR imaging technique.

## Supplementary Material

pgad070_Supplementary_Data

## Data Availability

The original data needed to evaluate the conclusions in the paper are available on Figshare (https://doi.org/10.6084/m9.figshare.22292716.v1) and/or the Supplementary Materials.
