# Supplementary material for: Pelagic microplastics in the North Pacific Subtropical Gyre: A prevalent anthropogenic component of the particulate organic carbon pool
Source: PNAS Nexus. 2023 Mar 9;2(3):pgad070. doi: 10.1093/pnasnexus/pgad070 (PMC10062330; doi:10.1093/pnasnexus/pgad070)
Supplement: pgad070_Supplementary_Data [file pgad070_supplementary_data.docx]

**Supporting Information for**

Pelagic microplastics in the North Pacific subtropical gyre: a prevalent anthropogenic component of the particulate organic carbon pool

Shiye Zhao^1^*, Tracy J. Mincer^2,3^*, Laurent Lebreton^4,5^, Matthias Egger^4,6^

^1^Japan Agency for Marine-Earth Science and Technology, 2-15 Natsushimacho, Yokosuka 237-0061, Japan

^2^Harbor Branch Oceanographic Institute, Florida Atlantic University, Fort Pierce, Florida, USA.

^3^Department of Biology, Wilkes Honors College, Florida Atlantic University, Jupiter, Florida, USA.

^4^The Ocean Cleanup, Rotterdam, The Netherlands

^5^The Modelling House, Raglan, New Zealand

^6^Egger Research and Consulting, St. Gallen, Switzerland

*Corresponding authors: [szhao@jamstec.go.jp](mailto:szhao@jamstec.go.jp), [tmincer@fau.edu](mailto:tmincer@fau.edu)

**This PDF file includes:**

Figures S1 to S10

Tables S1 to S7

SI References


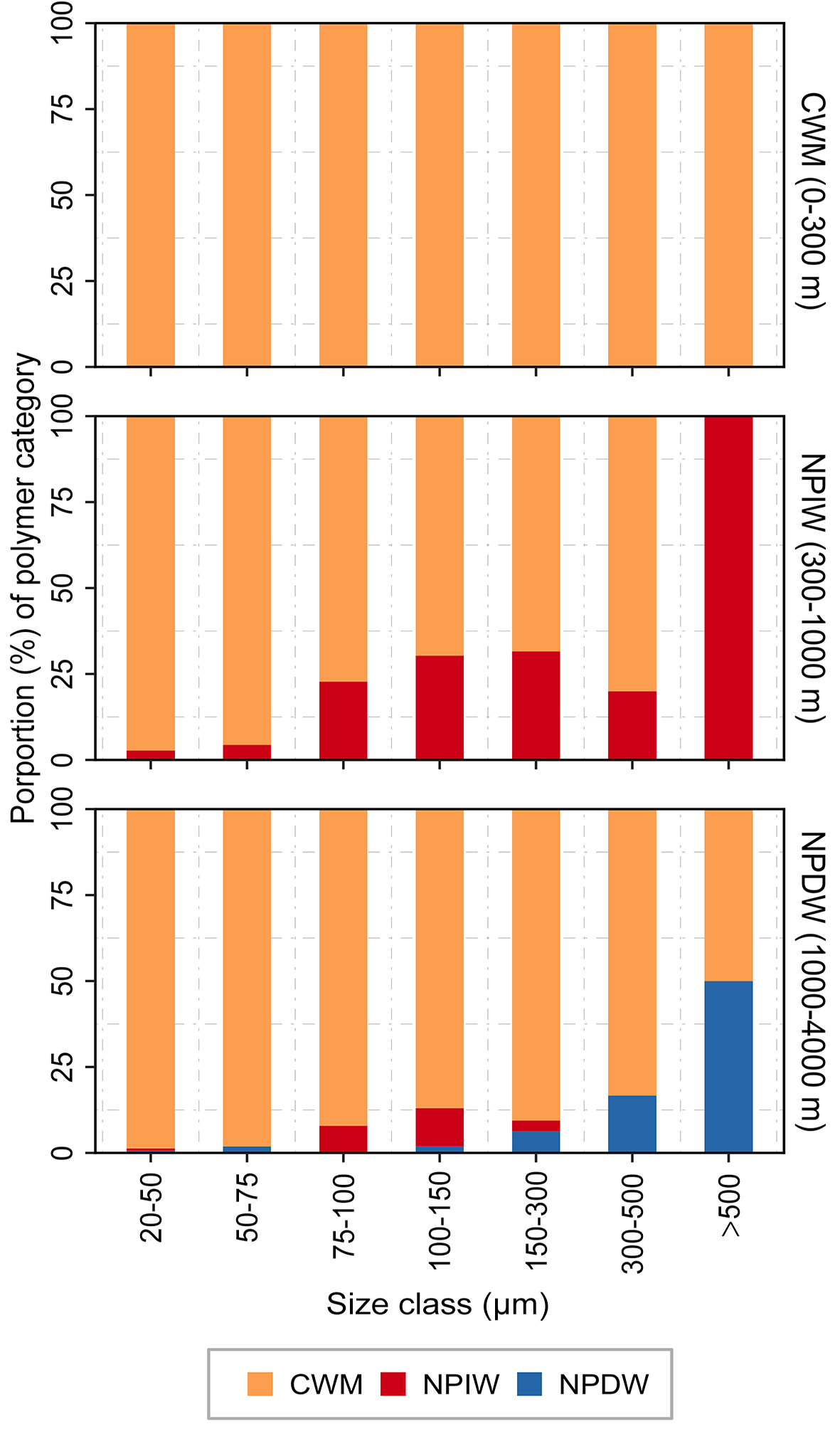


**Fig. S1.** Proportion (%) of polymers categorized as originating from the North Pacific Central Water Mass (CWM; 0–300 m; the upper panel), North Pacific Intermediate Water (NPIW; 300–1,000 m; the middle panel) and North Pacific Deep Water (NPDW; 1,000–4,000 m; the lower panel), in each water mass and each size class. Each category was defined as the water mass where they were first identitied, considering a directionality from suface to bathypelagic waters, and considering all three stations as a whole.


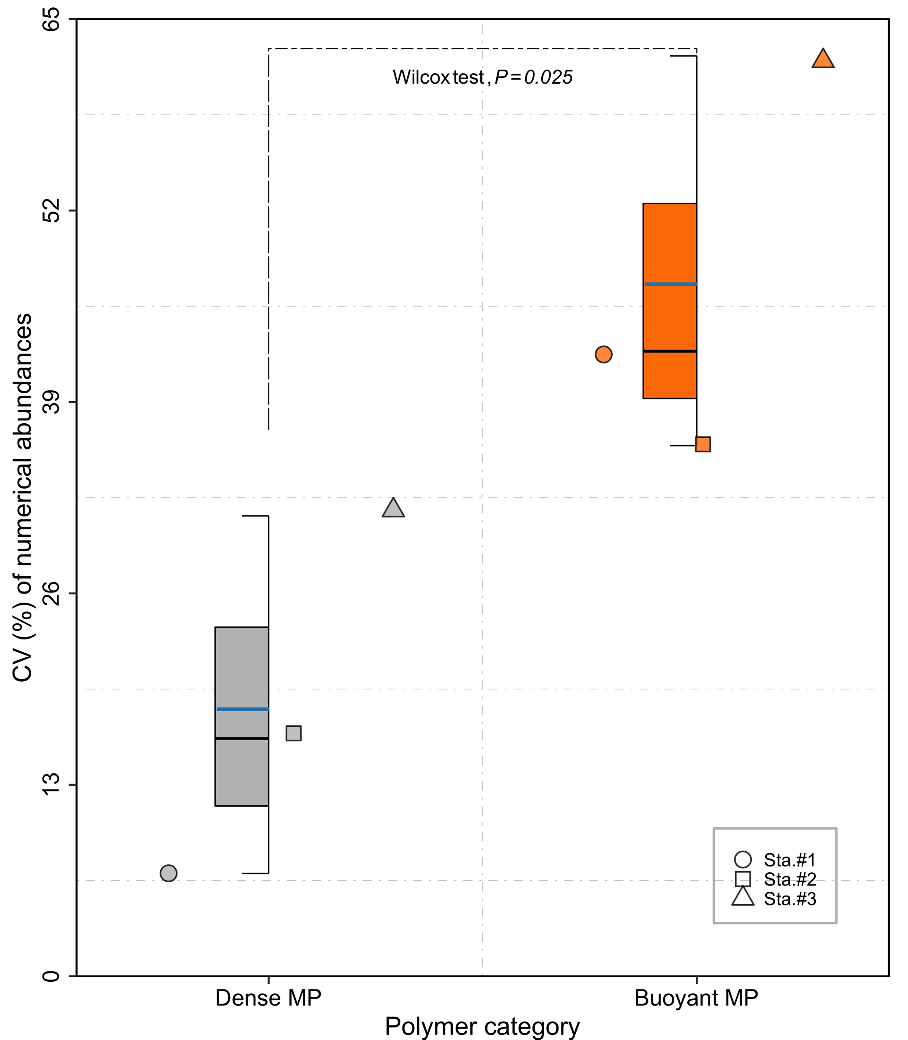


**Fig. S2.** Coefficient of variation (CV) of numerical abundances of dense and buoyant MP captured by *in-situ* pumps in the water column of the North Pacific subtropical gyre. Black and blue horizontal lines within the boxes represent boxplot medians and means. The top and bottom box boundaries of boxes represent the 25th and 75th percentiles; while whiskers indicate the largest and the smallest measured values within 1.5 interquartile ranges from the box.


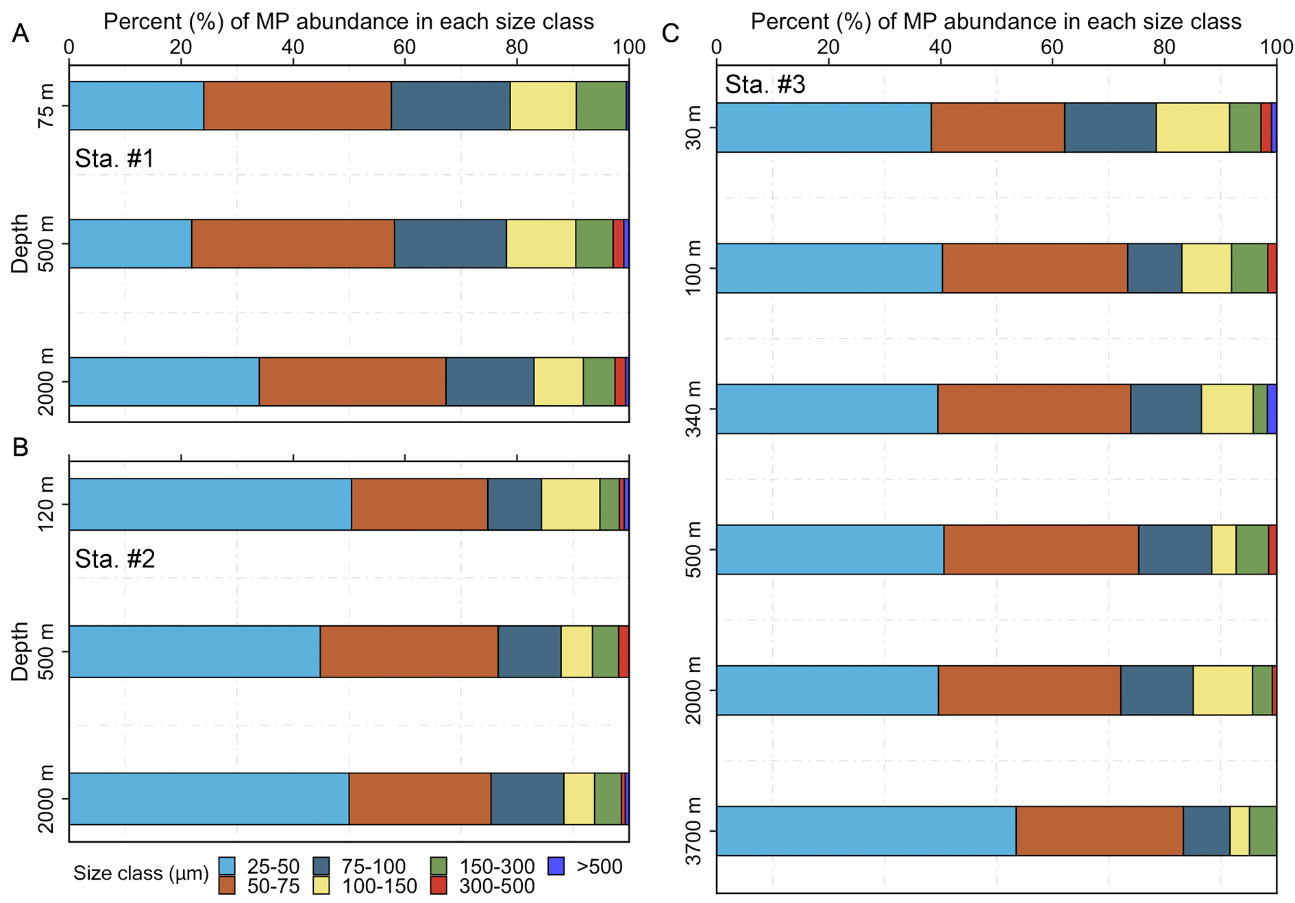


**Fig. S3.** Size fractions of MP numerical abundances at Sta. #1 (**A**), Sta. #2 (**B**) and Sta. #3 (**C**).


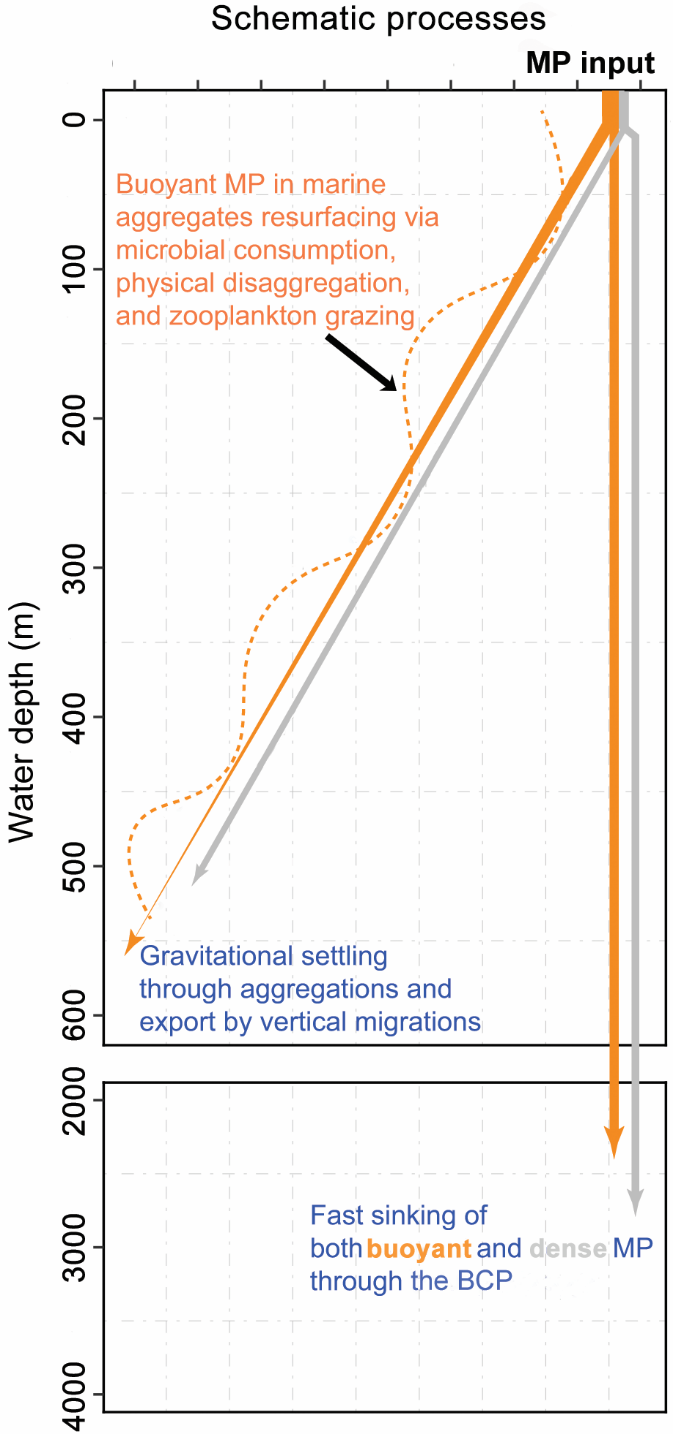


**Fig. S4.** Conceptual diagram illustrating the downward transport of MP. Orange and gray lines represent buoyant and dense MP, respectively. Our findings imply that MP in the water column is redistributed according to two frameworks: 1) Buoyant plastic particles, which are incorporated into marine snow, lose their buoyancy and sink out of the surface layer. During the downward transfer, MP escape from the biological pump processes via microbial consumption in aggregates, physical disaggregation and zooplankton grazing (orange dotted curved line), recover their buoyancy and resurface. This process results in an accumulation of buoyant MP in the upper portion of the mesopelagic zone; 2) Both buoyant and dense plastic particles integrated into marine aggregates and fecal pellets, the important components of biological carbon pump (BCP), are rapidly transported to the great depths.


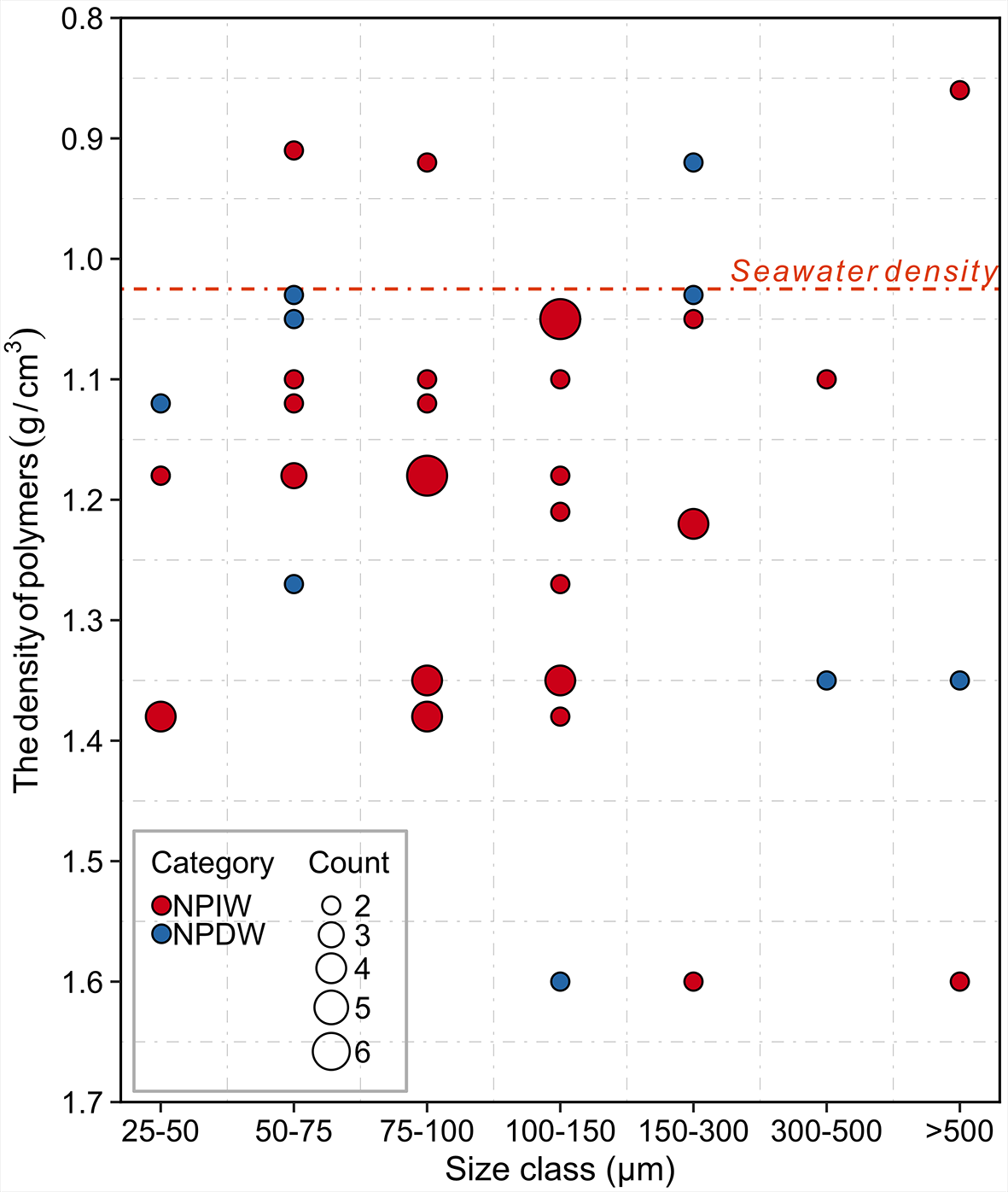


**Fig. S5.** The count (#), density (g/cm^3^) and size distribution of polymers first detected in the deep-water masses NPIW (North Pacific Intermediate Water: 300–1,000 m) and NPDW (North Pacific Deep Water: 1,000–4,000 m), respectively.


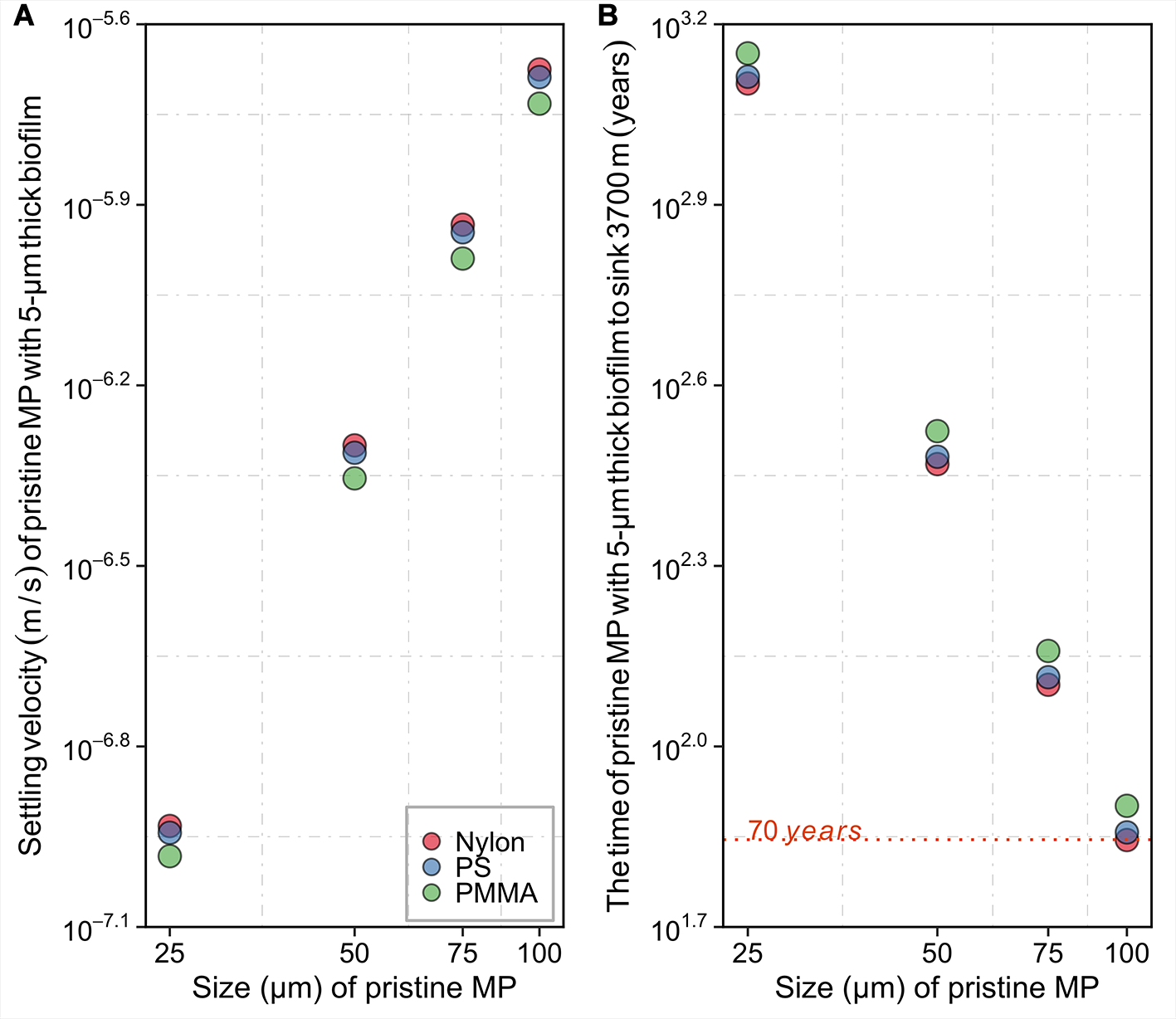


**Fig. S6.** The modeled settling velocities (A) and the time (B) to sink 3,700 m for the most common MP denser than seawater [Nylon; polystyrene, PS; poly(methyl methacrylate), PMMA]. It’s hypothesized that pristine MP with a size of 25, 50, 75 and 100 μm are all covered by 5-μm thick biofilm of diatoms, which have a density of 1.15 g/cm^3^. These three dense polymers account for 77.6% of the total dense MP counts (n=509 pieces).

*
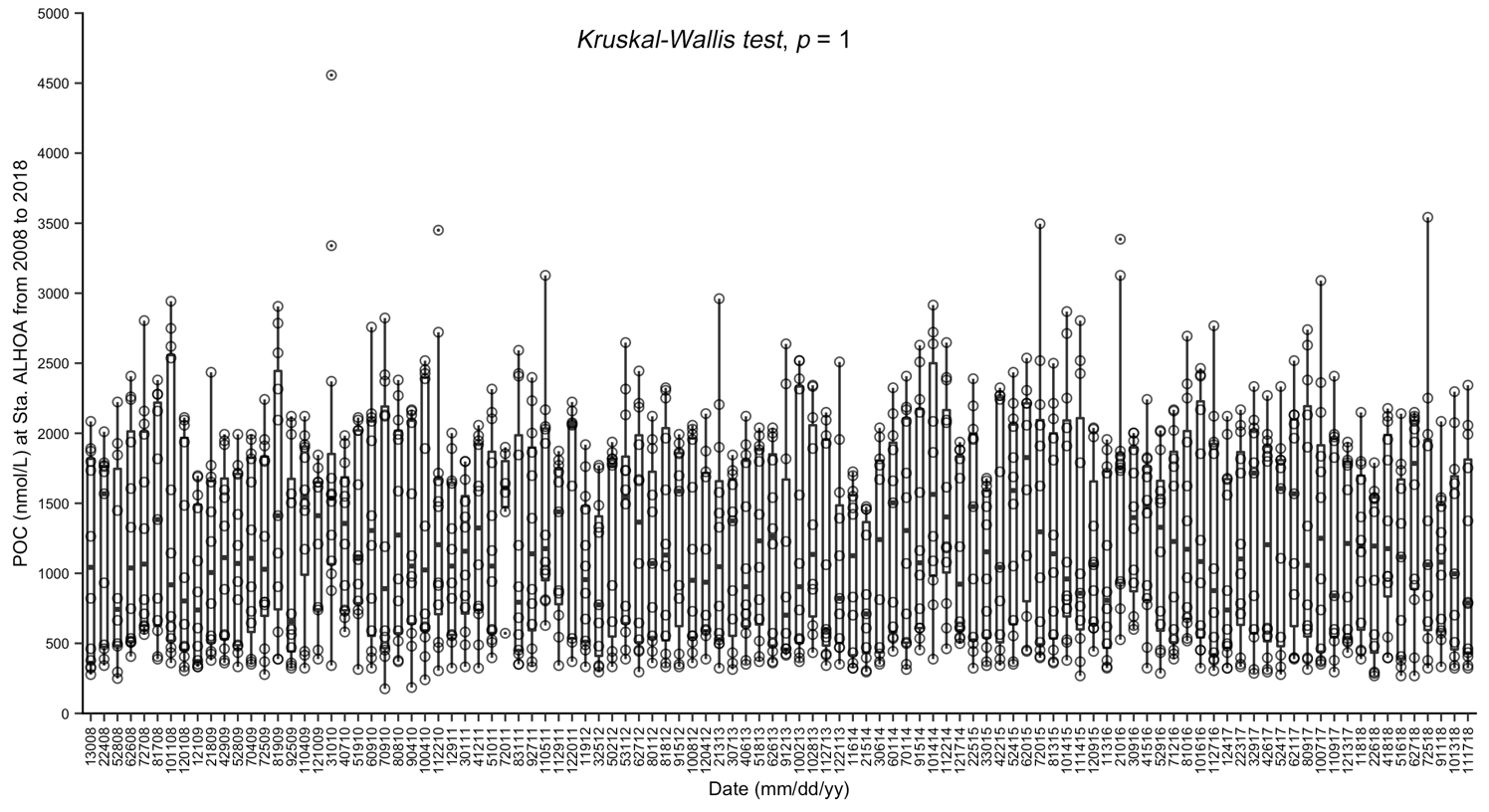
*

**Fig. S7.** The seasonal particulate organic carbon (POC) values measured at Sta. ALHOA from 2008 to 2018. No significant difference of POC values over the 10 years is detected (*Kruskal-Wallis* test, $\mathcal{X}^{2}$ = 55.6, *p* = 1).

*
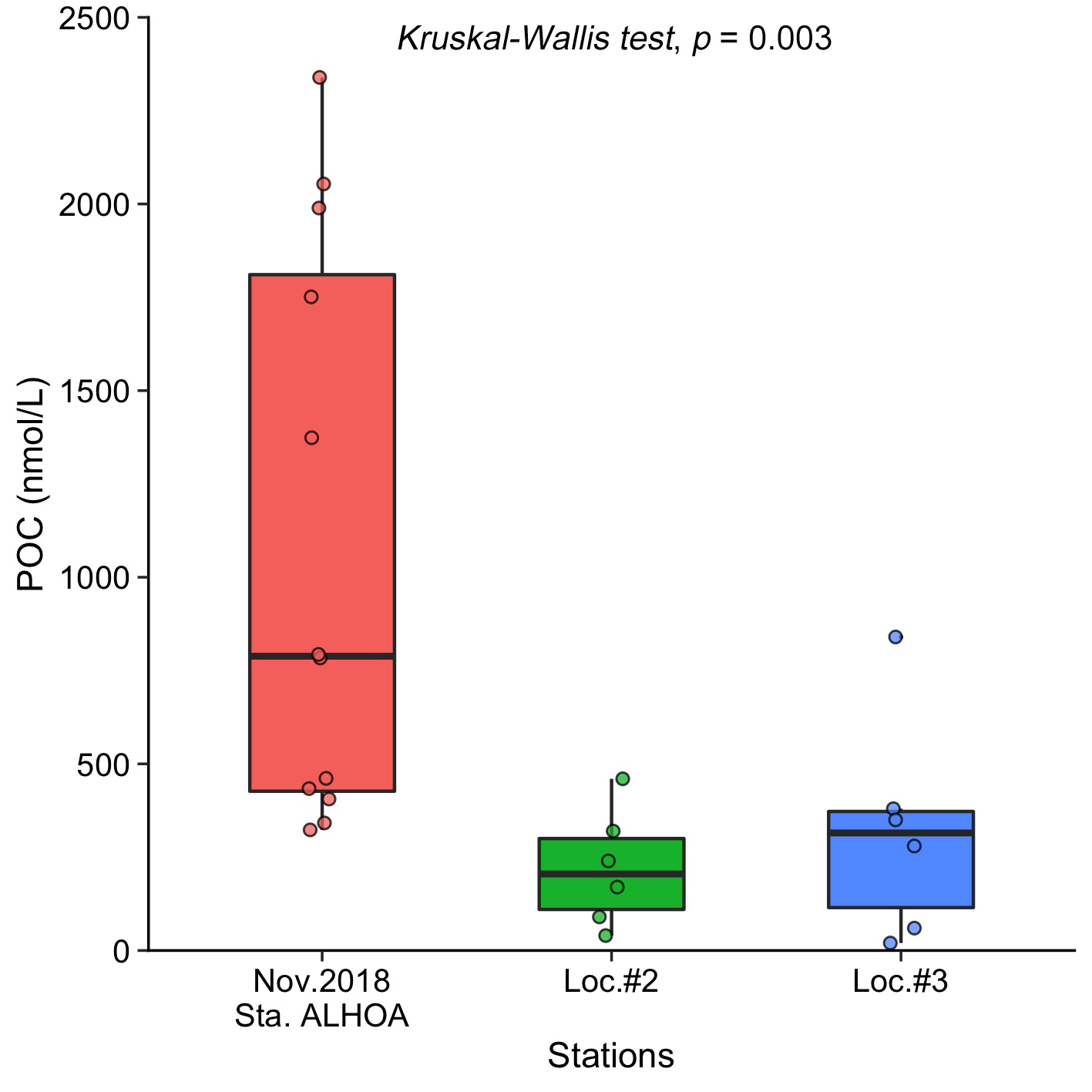
*

**Fig. S8.** Comparison of particulate organic carbon (POC) values between Sta. ALHOA, Loc.#2 and #3. Note that POC values at Sta. ALOHA are significantly higher than at stations Loc.#2 and Loc.#3 located in closer proximity to the North Pacific Garbage Patch (*Kruskal-Wallis* test, $\mathcal{X}^{2}$ = 11.1, *p* = 0.003).


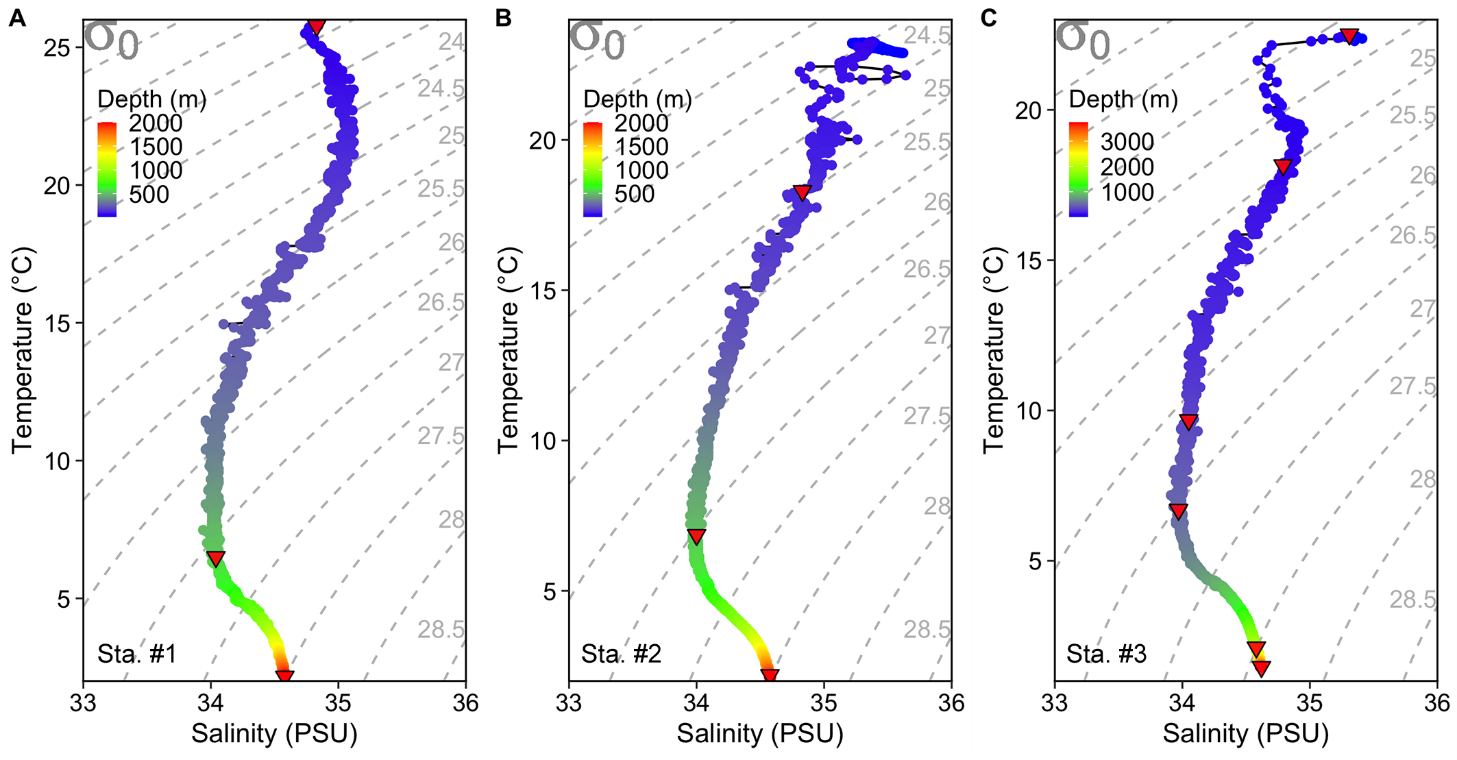


**Fig. S9.** The hydrographic properties of three sampling stations (A: Sta. #1; B: Sta. #2; C: Sta. #3).


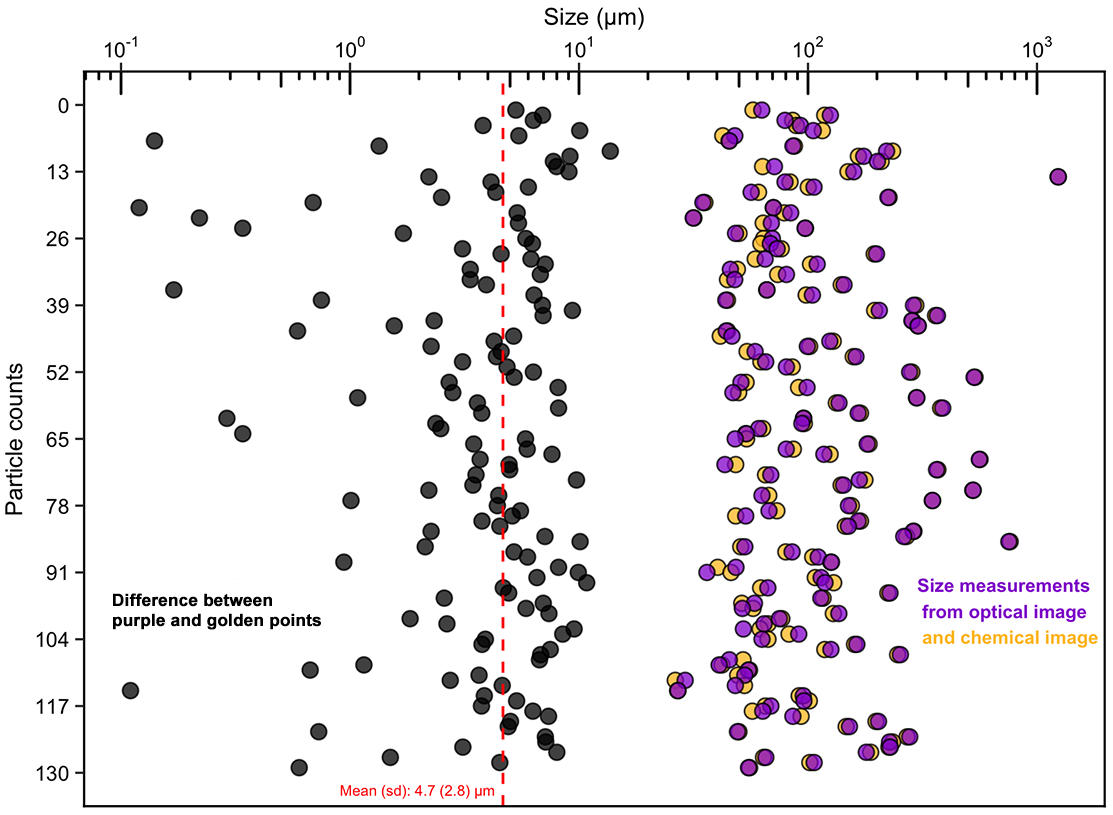


**Fig. S10.** The Ferret’s diameters of MP particle (130 pieces) measured on the infrared images (golden dots) and on the concurrent optical images (purple dots). Black dots represent the differences between two types of measurements. Red dash line indicates the averaged value of black dots.

**Table S1 Comparison of methodologies to quantify pelagic microplastics.**

| Study | **Study region** | **Sampling technique** | **Analytical Method** | **Targeted size fraction**  (μm) | **Reported**  **size range**  **(**μm) | **Targeted polymer types** | **Range of the sampling depths** |
| --- | --- | --- | --- | --- | --- | --- | --- |
| This study | NPSG | *in-situ* pump | µFTIR imaging | >10 | 25–1237 | All polymers | 30 m to 3700 m |
| Egger et al.^1^ | NPSG | Manta trawl | Raman and FTIR spectroscopes | >500 | 500–5000 | All polymers | Surface (0 to 0.15 m) |
|  |  | MOCNESS | Raman and FTIR spectroscopes | >500 | 500–5000 | All polymers | 2 m to 2000 m |
| Pabortsava and Lampitt^2^ | Atlantic Ocean | *in-situ* pump | µFTIR imaging | >55 | 32–651 | PE, PP, PS | 10 m to 200 m |
| Tekman et al.^3^ | Arctic Ocean | *in-situ* pump | µFTIR imaging | >11 | 11–200 | All polymers | 1 m to 5569 m |
| Zhao et al.^4^ | SASG | *in-situ* pump, | µFTIR imaging | 2-40 | 20–321 | All polymers | 10 m to 5200 m |
|  |  | Manta trawl | ATR-FTIR spectroscope | >500 | Not provided | All polymers | 20 cm |
|  |  | MultiNet | Density floating and visual inspection | >200 | Not provided | All polymers | 5 m to 100 m |
| Egger et al.^5^ | NASG | Manta trawl | Raman and FTIR spectroscopes | >500 | 500–5000 | All polymers | Surface (0 to 0.15 m) |
|  |  | MultiNet | Raman and FTIR spectroscopes | >500 | 500–5000 | All polymers | 5 m to 300 m |
| Galgani et al et al.^6^ | NASG | Sediment trap | µFTIR imaging | >20 | >20 | All polymers | 50 m to 600 m |
| Galgani et al et al.^6^ | NASG | Sediment trap | Py-GC/MS | >10 | >10 | All polymers | 50 m to 600 m |

Notes: North Pacific subtropical gyre, NSPG; South Atlantic subtropical gyre, SASG; North Atlantic subtropical gyre (NASG), polyethylene, PE; polypropylene, PP; polystyrene, PS; micro-Fourier Transform Infrared, µFTIR; Pyrolysis Gas Chromatography coupled with Mass Spectrometry, Py-GC/MS

**Table S2. Sampling information and plastic counts confirmed in this study.**

| Station | GPS Coordinates | Depth (m) | Water mass | Filtered volume (L) | Plastic counts (n) | | |
| --- | --- | --- | --- | --- | --- | --- | --- |
|  |  |  |  |  | Samples | Field blanks | Laboratory procedural blanks |
| #1 | 151.902W | 75 | CWM | 405 | 212 | 4 | 0 |
|  | 25.218N | 500 | NPIW | 408 | 105 | 5 |  |
|  |  | 2,000 | NPDW | 412 | 159 | 6 |  |
| #2 | 147.019W | 120 | CWM | 510 | 115 | — | 1 |
|  | 28.601N | 500 | NPIW | 397 | 107 | — |  |
|  |  | 2,000 | NPDW | 411 | 146 | 18 |  |
| #3 | 145.026W | 30 | CWM | 450 | 214 | — | 1 |
|  | 30.534N | 100 | CWM | 459 | 124 | — |  |
|  |  | 340 | NPIW | 492 | 119 | 7 |  |
|  |  | 500 | NPIW | 470 | 69 | — |  |
|  |  | 2,000 | NPDW | 458 | 255 | — |  |
|  |  | 3,700 | NPDW | 477 | 144 | — |  |
| Total |  |  |  |  | 1,769 | 40 | 2 |

Notes: North Pacific Central Water Mass (0–300 m), CWM; North Pacific Intermediate Water (300–1,000 m), NPIW; North Pacific Deep Water (1,000–4,000 m), NPDW. Field blank filters (142-mm stainless steel meshes; n=5) were simultaneously deployed with the in-situ pump at certain depths of each station by specially disconnecting one of the dual filter holders from pumped water flow. The procedure blank was performed by exposing a pre-combusted 25 mm-Petri dish to the air under the laminar flow cabinet. Then, the potential particles collected in the petri dish were processed following the identical procedure as for the pump samples and scanned with the μFTIR imaging technique.

**Table S3. Abundances of MP measured in this study and predicted large MP (>500 μm) abundances at the sampling depths using the power law functions provided by Egger et al.^1^.**

| Station | Depth (m) | MP abundance (*in situ* pump) | | Predicted large MP abundance | | Total abundance | |
| --- | --- | --- | --- | --- | --- | --- | --- |
|  |  | #/m^3^ | μg/m^3^ | #/m^3^ (×10^-4^) | μg/m^3^ | #/m^3^ | μg/m^3^ |
| **#1** | 75 | 524 | 48.0 | 12.7 | 3.4 | 524 | 51.5 |
|  | 500 | 258 | 24.5 | 1.8 | 0.5 | 258 | 25.0 |
|  | 2000 | 386 | 31.3 | 0.4 | 0.1 | 386 | 31.3 |
| **#2** | 120 | 226 | 14.8 | 15.1 | 4.5 | 226 | 19.2 |
|  | 500 | 269 | 16.0 | 4.7 | 1.1 | 269 | 17.1 |
|  | 2000 | 355 | 10.9 | 1.5 | 0.3 | 355 | 11.2 |
| **#3** | 30 | 475 | 29.4 | 187.1 | 39.3 | 475 | 68.7 |
|  | 100 | 270 | 11.3 | 55.5 | 8.9 | 270 | 20.2 |
|  | 340 | 242 | 11.5 | 16.1 | 2.0 | 242 | 13.4 |
|  | 500 | 147 | 12.9 | 10.9 | 1.2 | 147 | 14.1 |
|  | 2000 | 557 | 28.5 | 2.7 | 0.2 | 557 | 28.7 |
|  | 3700 | 302 | 10.7 | 1.5 | 0.1 | 302 | 10.8 |

Notes: “Predicted large MP abundance” was predicted using the power law models for each station as provided by Egger et al.^1^ (2020) (see also Table S4). The total abundance is the sum of ‘Predicted large MP abundance’ and MP abundance measured in the in-situ pump samples in this study.

**Table S4. Linear regressions relationships between water depth and the ratios of small MP (>25 μm, collected by *in-situ* pumps) and large MP [>500 μm, predicted according to the models in** Egger et al. (2020)^1^**] abundances.**

| Station | R^2^ | p | Model | Ratio Type |
| --- | --- | --- | --- | --- |
| Sta.#1 | 0.9605 | 0.001 | $\log y=1.05\log x-3.9$ | Numerical abundance |
| Sta.#2 | 0.9995 | 0.01 | $\log y=1.03\log x-3.2$ | Numerical abundance |
| Sta.#3 | 0.9332 | 0.142 | $\log y=0.93\log x-2.4$ | Numerical abundance |
| Sta.#1 | 0.9712 | 0.1086 | $\log y=1.05\log x-3.9$ | Mass abundance |
| Sta.#2 | 0.9888 | 0.06 | $\log y=1.03\log x-3.2$ | Mass abundance |
| Sta.#3 | 0.9541 | 0.0008 | $\log y=0.93\log x-2.4$ | Mass abundance |

**Notes:** $y$ represents the water depth (m)**;** $x$indicates the ratio of small MP/large MP numerical (#/m^3^) or mass abundances (μg/m^3^).

**Table S5. Theoretical density and chemical formulas of polymer types identified.**

| **No** | **Polymer type** | **Density (g/cm^3^)** | **Chemical formula** | **Carbon portion (%)** | **Polymer category** |
| --- | --- | --- | --- | --- | --- |
| 1 | Polyethylene | 0.92 | C2H4 | 85.6 | Buoyant polymer |
| 2 | Poly(Ethylene:Propylene) | 0.9 | C2H4-C3H6 | 85.6 | Buoyant polymer |
| 3 | Poly(Ethylene:Propylene:Diene) | 0.86 | C2H4-C3H6-C9H12 | 86 | Buoyant polymer |
| 4 | Polypropylene | 0.86 | C3H6 | 85.6 | Buoyant polymer |
| 5 | Nylon 6/6.6 | 1.22 | C12H22N2O2 | 46.8 | Dense polymer |
| 6 | Polystyrene | 1.05 | C8H8 | 92.3 | Dense polymer |
| 7 | Poly(Methyl Methacrylate) | 1.18 | C5O2H8 | 60 | Dense polymer |
| 8 | Polyester | 1.35 | C10H8O4 | 62.5 | Dense polymer |
| 9 | Poly(Vinyl Chloride) | 1.38 | C2H3Cl | 38.4 | Dense polymer |
| 10 | Polyetherurethane | 1.12 | C3H8N2O | 41 | Dense polymer |
| 11 | Poly(Vinylidene Fluoride:C3F6) | 1.78 | C5H2F8 | 44.8 | Dense polymer |
| 12 | Poly(Ethyl Acrylate) | 1.05 | C5H8O2 | 60 | Dense polymer |
| 13 | Alkyd Resin (Paint) | 1.6 |  | 62.5 | Dense polymer |
| 14 | Poly(Butadiene) | 0.91 |  | 62.5 | Buoyant polymer |
| 15 | Poly(Ethylene:Vinyl Chloride) | 1.04 |  | 62.5 | Dense polymer |
| 16 | Urethane Alkyd | 0.92 |  | 62.5 | Buoyant polymer |
| 17 | Poly(Vinyl Chloride:MMA) | 1.21 |  | 62.5 | Dense polymer |
| 18 | Poly(Styrene:Acrylonitrile:MMA) | 1.18 |  | 62.5 | Dense polymer |
| 19 | Aromatic Hydrocarbon Resin | 1.1 |  | 62.5 | Dense polymer |
| 20 | Poly(Acrylonitrile:MMA) | 1.18 |  | 62.5 | Dense polymer |
| 21 | Poly(Methylphenylsiloxane) | 1.1 |  | 62.5 | Dense polymer |
| 22 | Poly(Vinyl Acetate:Ethylene) | 0.95 |  | 62.5 | Buoyant polymer |
| 23 | Poly(Butadiene:MMA) | 1.03 |  | 62.5 | Dense polymer |
| 24 | Poly(Trimellitamide Imide) | 1.27 |  | 62.5 | Dense polymer |
| 25 | Poly(Ethylacrylate:ST:Acrylamide) | 1.05 |  | 62.5 | Dense polymer |

Notes: ‘62.5’ is the median carbon portion of twelve polymer types from No.1-12. The density is sourced from the Polymer Database (CROW logo Polymer Science). Polymers with theoretical densities lower than 1.025 g/cm^3^ are regarded as buoyant plastics, while polymer species (ρ >1.025 g/cm^3^) are referred to as dense plastics.

**Table S6. Particle size distributions of MP particles in the eastern North Pacific subtropical gyre.**

| **Study** | **Location** | **Station** | **Depth (m)** | **PSD Slope (ξ)** | ***R^2^*** | ***P*** |
| --- | --- | --- | --- | --- | --- | --- |
| This study | North Pacific subtropical gyre | #1 | 75 | 1.54 | 0.99 | 0.008 |
|  |  |  | 500 | 1.77 | 0.99 | 0.006 |
|  |  |  | 2,000 | 2.10 | 0.99 | 0.001 |
| This study |  | #2 | 120 | 2.11 | 0.96 | 0.001 |
|  |  |  | 500 | 2.80 | 0.99 | 0.011 |
|  |  |  | 2,000 | 2.10 | 0.99 | 0.003 |
| This study |  | #3 | 30 | 1.31 | 0.99 | 0.004 |
|  |  |  | 100 | 1.31 | 0.85 | 0.001 |
|  |  |  | 340 | 2.54 | 0.99 | 0.014 |
|  |  |  | 500 | 2.85 | 0.98 | 0.005 |
|  |  |  | 2,000 | 2.23 | 0.99 | 0.001 |
|  |  |  | 3,700 | 2.64 | 0.98 | 0.099 |

**Table S7. Power law functions between water depth and particulate organic carbon (POC) as observed at three stations from the eastern North Pacific subtropical gyre.**

| Station | Location | R^2^ | p | Model | Reference |
| --- | --- | --- | --- | --- | --- |
| Loc.#2 | 27.75N, 155.28W | 0.7319 | 0.01 | $y=7.4-2.1x$ | Subhas et al.^7^ |
| Loc.#3 | 35.26N, 150.98W | 0.957 | 0.0004 | $y=5.1-1.1x$ | Subhas et al.^7^ |
| ALOHA | 23.5N, 158W | 0.7376 | 0.0002 | $y=7.4-1.8x$ | ALOHA^8^ |

Notes: $y={Log}_{10}water depth$, $x={Log}_{10}POC concentration$

**SI References**

1 Egger, M., Sulu-Gambari, F. & Lebreton, L. First evidence of plastic fallout from the North Pacific Garbage Patch. *Scientific Reports* **10**, 1-10 (2020).

2 Pabortsava, K. & Lampitt, R. S. High concentrations of plastic hidden beneath the surface of the Atlantic Ocean. *Nature Communications* **11**, 1-11 (2020).

3 Tekman, M. B. *et al.* Tying up loose ends of microplastic pollution in the Arctic: distribution from the sea surface through the water column to deep-sea sediments at the HAUSGARTEN Observatory. *Environmental Science & Technology* **54**, 4079-4090 (2020).

4 Zhao, S. *et al.* Large quantities of small microplastics permeate the surface ocean to abyssal depths in the South Atlantic Gyre. *Global Change Biology* **28**, 2991-3006 (2022).

5 Egger, M. et al. Pelagic distribution of plastic debris (> 500 µm) and marine organisms in the upper layer of the North Atlantic Ocean. *Scientific Reports* **12**, 13465 (2022).

6. Galgani L. et al. Hitchhiking into the deep: how microplastic particles are exported through the biological carbon pump in the North Atlantic Ocean. Environmental Science & Technology (2022). DOI: 10.1021/acs.est.2c04712

7 Subhas, A. V., Adkins, J. F., Dong, S., Rollins, N. E. & Berelson, W. M. The carbonic anhydrase activity of sinking and suspended particles in the North Pacific Ocean. *Limnology and Oceanography* **65**, 637-651 (2020).

8 ALOHA. (http://hahana.soest.hawaii.edu/hot/hot_jgo fs.html). (2018).
